# *Lactobacillus fermentum* ZYL0401 Attenuates Lipopolysaccharide-Induced Hepatic TNF-α Expression and Liver Injury via an IL-10- and PGE_2_-EP4-Dependent Mechanism

**DOI:** 10.1371/journal.pone.0126520

**Published:** 2015-05-15

**Authors:** Pengfeng Jin, Yunbo Chen, Longxian Lv, Jianzhuan Yang, Haifeng Lu, Lanjuan Li

**Affiliations:** 1 State Key Laboratory for Diagnosis and Treatment of Infectious Disease, The First Affiliated Hospital, Zhejiang University, Hangzhou, China; 2 Collaborative Innovation Center for Diagnosis and Treatment of Infectious Diseases, Hangzhou, China; University of Louisville School of Medicine, UNITED STATES

## Abstract

Lipopolysaccharide (LPS) has essential role in the pathogenesis of D-galactosamine-sensitized animal models and alcoholic liver diseases of humans, by stimulating release of pro-inflammatory mediators that cause hepatic damage and intestinal barrier impairment. Oral pretreatment of probiotics has been shown to attenuate LPS-induced hepatic injury, but it is unclear whether the effect is direct or due to improvement in the intestinal barrier. The present study tested the hypothesis that pretreatment with probiotics enables the liver to withstand directly LPS-induced hepatic injury and inflammation. In a mouse model of LPS-induced hepatic injury, the levels of hepatic tumor necrosis factor-alpha (TNF-α) and serum alanine aminotransferase (ALT) of mice with depleted intestinal commensal bacteria were not significantly different from that of the control models. Pre-feeding mice for 10 days with *Lactobacillus fermentum* ZYL0401 (LF41), significantly alleviated LPS-induced hepatic TNF-α expression and liver damage. After LF41 pretreatment, mice had dramatically more *L*.*fermentum*-specific DNA in the ileum, significantly higher levels of ileal cyclooxygenase (COX)-2 and interleukin 10 (IL-10) and hepatic prostaglandin E2 (PGE_2_). However, hepatic COX-1, COX-2, and IL-10 protein levels were not changed after the pretreatment. There were also higher hepatic IL-10 protein levels after LPS challenge in LF41-pretreaed mice than in the control mice. Attenuation of hepatic TNF-α was mediated via the PGE_2_/E prostanoid 4 (EP4) pathway, and serum ALT levels were attenuated in an IL-10-dependent manner. A COX-2 blockade abolished the increase in hepatic PGE_2_ and IL-10 associated with LF41. In LF41-pretreated mice, a blockade of IL-10 caused COX-2-dependent promotion of hepatic PGE_2_, without affecting hepatic COX-2levels. In LF41-pretreated mice, COX2 prevented enhancing TNF-α expression in both hepatic mononuclear cells and the ileum, and averted TNF-α-mediated increase in intestinal permeability. Together, we demonstrated that LF41 pre-feeding enabled the liver to alleviate LPS-induced hepatic TNF-α expression and injury via a PGE_2_-EP4- and IL-10-dependent mechanism.

## Introduction

Lipopolysaccharides (LPS), produced by Gram-negative bacteria, enter the systemic circulation and activate the innate immune system. This leads to the secretion of pro-inflammatory cytokines, which have important pathogenic role in acute and chronic liver diseases [1]. In nonalcoholic steatohepatitis, LPS promote the production of tumor necrosis factor alpha (TNF-α) and other pro-inflammatory cytokines [2]. In alcoholic liver disease, gut-derived LPS stimulate hepatic macrophages to produce TNF-α, interleukin-6 (IL-6), and interleukin-1β (IL-1β), which exacerbate liver injury [3–5]. Furthermore, pro-inflammatory cytokines elicited by LPS, such as TNF-α, have been positively associated with severity of disease in patients with severe alcoholic liver disease [6]. In models of liver disease sensitized by D-galactosamine (GalN), TNF-α derived from LPS-activated Kupffer cells accelerates hepatocyte apoptosis under the assistance of GalN, leading to release of large quantities of harmful mediators that can aggravate liver damage through impairment of gut barrier [7–8].

Probiotics are the living microorganisms that can confer a health benefit to the host should they be ingested in adequate amounts [9]. In recent years, there has been increased interest in the immunomodulatory functions of probiotics. Such activities include maintaining gut microbial homeostasis and the gastrointestinal barrier function, antagonizing the colonization of pathogens, and the regulation of local and systemic immune responses [10].

Several probiotics have been shown to have protective effect against some hepatic diseases associated with LPS. For example, patients with alcoholic liver cirrhosis were shown to have significantly improved liver function and lower plasma levels of TNF-α and IL-6 after oral supplementation with probiotic formula VSL#3 [11]. Pretreatment with VSL#3 was also associated with lower levels of hepatic pro-inflammatory cytokines induced by GalN/LPS, and less liver damage and dysfunction of the gut barrier [12]. Pretreatment withVSL#3 may promote the entrance of gut-derived factor(s) into the liver, to up-regulate hepatic peroxisome proliferator-activated receptor gamma, a nuclear receptor whose antagonism to LPS-induced inflammation is well known [12–14]. It has also been shown in a murine alcoholic liver disease model that oral administration of supernatant derived from *Lactobacillus rhamnosus* GG (LGG) correlates with attenuation of the hepatic inflammation and injury and intestinal barrier dysfunction [15]. In addition, several other probiotics have been associated with amelioration of hepatic injury and pro-inflammatory responses, disturbance of the intestinal microbiota, and intestinal barrier dysfunction in GalN-sensitized animal models [16–18].

The improvement in hepatic function associated with these probiotics in GalN-sensitized and alcoholic liver disease models is usually attributive to their direct precautionary action against the impairment of intestinal barrier, thus alleviating the aggravation of these diseases [12, 15–18]. However, there is a close vascular and lymphatic link between the liver and gut, and orally administered probiotics are able to influence local and systemic immunity in experimental animals and humans [10]. Therefore, we hypothesized that oral pretreatment of these probiotics might enable the liver to directly attenuate pro-inflammatory responses and liver damage induced by subsequent LPS stimulation. However, when using GalN-sensitized or alcoholic liver disease model, it is difficult to isolate the direct effect of oral probiotics on the liver from the indirect one that is resulted from reducing transfer of intestine-derived LPS to the liver [1, 8].

The present study used a mouse model of LPS-induced hepatic injury to investigate the direct effect of orally administered probiotic strains on hepatic inflammation and damage. We first showed that the levels of hepatic TNF-α and serum alanine aminotransferase (ALT) of model animals with depleted intestinal commensal bacteria were not significantly different from that of the control models. Then, several probiotic strains that had been previously shown to exert preventive action against the hepatic injury in GalN-sensitized and alcoholic liver disease models were administered as oral pretreatment. We demonstrated here that pre-feeding mice with *Lactobacillus fermentum* ZYL0401 (LF41) [16] for 10 days significantly attenuated LPS-induced hepatic TNF-α expression and liver injury. LF41 pretreatment significantly increased hepatic prostaglandin E_2_ (PGE_2_) levels, without affecting both hepatic cyclooxygenase (COX)-2 and COX-1 expression, and augmented LPS-activated hepatic IL-10 levels. The preventive effect against hepatic TNF-α expression was mediated via the PGE_2_/E prostanoid 4 (EP4) pathway, and serum ALT levels was suppressed in an IL-10-dependent manner.

## Materials and Methods

### Ethics Statement

Experiments and animal care were performed in accordance with protocols approved by the Zhejiang University Institutional Animal Care and Use Committee.

### Bacterial Preparation

Two strains from our laboratory, *Lactobacillus fermentum* ZYL0401 (LF41) and *Bifidobacterium catenulatum* ZYB0401 (BC41) [16], and *Lactobacillus rhamnosus* GG (LGG; ATCC 53103), were selected in our studies. For preparation of the bacteria-derived conditioned media needed in some of the *in vitro* experiments, each strain was diluted in de Man, Rogosa, and Sharpe (MRS) medium (Difco, BD, Sparks, MD) at different OD_600_ values and cultured anaerobically at 37°C for 24 hours. These cultures were spun at 4500RCF for 8 minutes and the supernatants were filtered through a polyvinylidene fluoride membrane as conditioned media.

For *in vivo* experiments, all bacterial strains were cultured for 24 hours, collected, and re-suspended in phosphate-buffered saline (PBS) for subsequent oral administration.

### Depletion of Gut Commensal Microflora

Mice were given ampicillin (1 g/L; Sigma,St. Louis, MO), vancomycin (500 mg/L; Sigma), neomycin sulphate (1 g/L; Sigma), and metronidazole (1 g/L; Sigma) in drinking water for 4 weeks as described [19]. Mice were switched to water without antibiotics for 3 days before experimental manipulation. Bacterial load in the feces was quantified after the depletion by isolating DNA and performing quantitative PCR for total bacterial 16S rRNA genes.

### Animals and Experimental Protocol

C57BL/6 female and male mice (6 to 8 wk old) were purchased from Shanghai Slac Animal (Shanghai, China).

In the first round of experiments, groups of mice were given 10 consecutive days of daily intragastric (IG) supplements of 500 μL PBS containing: 1 × 10^9^ colony-forming units (cfu) LF41 (L-LF41); 2 × 10^10^ cfu LF41 (H-LF41); 2 × 10^10^ UV-killed cfu LF41 (killed-LF41); 2 × 10^10^ cfu LGG (LGG group); 2 × 10^10^ cfu BC41 (BC41 group); or PBS (control). These pretreated-mice were subsequently given one intraperitoneal (IP) injection of LPS (500 μg/kg body weight in 100 μL PBS; from *Escherichia coli* 0111:B4; Sigma) or 100 μL PBS. In the second round of experiments, groups of mice were given 21 consecutive days of daily IG supplements of 500 μL PBS containing 2 × 10^10^ cfu LF41 or PBS, and then one intraperitoneal (IP) injection of LPS (500 μg/kg body weight in 100 μL PBS) or 100 μL PBS. In the third round of experiments, mice with their intestinal commensal bacteria depleted through antibiotic ingestion (described above), or not (control) were given IP inoculation of 100 μL either PBS or LPS (500 μg/kg body weight).

Mice were killed by cervical dislocation either after only bacterial pretreatment or at different time points after LPS treatment for determination of experimental parameters described later. Liver samples were obtained and frozen at—80°C. Tissues from diverse segments of the gastrointestinal tract, including distal jejuna, terminal ileum, and proximal colon were quickly removed and washed with ice-cold PBS supplemented with penicillin, streptomycin, and fungizone (P/S/F, 100 U/mL; Sigma) before storage at—80°C. In some cases, ileal epithelial cells, lamina propria cells, and hepatic mononuclear cells were isolated. These collected tissues and cells were used to perform experimental tests, such as extraction of whole RNA and protein for evaluation of cytokines production. The tissues to be used for gene expression analysis were stored in the RNA stabilization reagent, *RNAlater* (Qiagen,Hilden, Germany), for RNA extraction.

### Cell Isolation

Isolation of the epithelial cells and lamina propria cells from the ileum was performed as previously described [20], with slight modification. Briefly, with the mice under anesthesia the abdomens were opened by a midline incision. Terminal ileums were then quickly removed and placed on a sterile plastic plate sitting on ice. The segment was flushed 5 times with ice-cold PBS containing antibiotics P/S/F (100 U/mL), opened longitudinally, and cut into 2- to 3-mm fragments. These fragments were then set in ice-cold RPMI 1640 medium (Gibco, Shanghai, China) supplemented with 5% fetal bovine serum (FBS) (Gibco), dithiothreitol (1 mmol/L), and P/S/F. After shaking vigorously, the supernatant was discarded and the tissue was incubated in RPMI 1640 with 5% FBS containing ethylenediaminetetraacetic acid (EDTA; 1 mmol/L) and P/S/F at 37°C for 20 minutes with 250-rpm agitation. After the incubation, the supernatant was transferred to centrifuge tubes and centrifuged for 3 min at 300 rpm and the pallet was harvested as ileal epithelial cells. The remained tissue was further incubated in RPMI 1640 with 5% FBS containing collagenase type VIII (Sigma; 1 mg/mL) and P/S/F for 30 minutes at 37°C with shaking (250 rpm). The cell suspension was sieved through a cell strainer (100 μm; BD Bioscience Pharmingen, San Diego, CA), and lamina propria cells were collected by centrifugation for 3 min at 300 rpm.

To isolate total hepatic mononuclear cells (HMNCs), whole liver tissues from killed mice were immediately removed and minced into small pieces. The pieces were shaken in the digestion buffer at 37°C for 25 min, homogenized and filtered through a 70-μm cell strainer (BD bioscience). To eliminate of hepatocytes, the cell suspension was centrifuged at 400 rpm for 5 min at room temperature. The supernatant was collected, washed in PBS and resuspended in a 40% Percoll gradient (GE healthcare). The cell suspension was gently overlaid onto 70% Percoll and centrifuged at 2,400 rpm for 30 min. HMNCs were collected from the interface.

These isolated cells were used to extract total RNA for determination of gene expression by quantitative real-time PCR and to quantify protein by western blot assay, detailed in other sections.

### Liver Function Tests

Liver Function Tests were performed as previously described in our laboratory [16]. Briefly, serum was obtained from whole blood samples centrifuged at 3,000 *× g* for 10 minutes at room temperature. Serum alanine aminotransferase (ALT) levels were assessed using a Hitachi 7600 automatic analyzer (Hitachi, Tokyo, Japan).

### Histologic Evaluation

Liver tissues were fixed in formalin, paraffin-embedded, cut into 4-μm sections, and then examined under light microscopy after staining with hematoxylin and eosin. Inflammatory foci are arbitrarily defined as collections of ≥5 leukocytes in the field (objective × 40). At least three slides were studied from each specimen in a blinded fashion.

### Enzyme-Linked Immunosorbent Assay (ELISA)

TNF-α protein concentration in the serum were determined using quantitative enzyme-linked immunosorbent assay kits (R&D Systems, MI) in accordance with the manufacturer’s recommended protocol.

For evaluation of PGE_2_ and TNF-α secretion in terminal ileum of mice, the tissues were removed, opened longitudinally, washed with cold PBS supplemented with antibiotics P/S/F (100 U/ml), and kept in cold serum-free RPMI 1640 medium (Gibco) supplemented with P/S/F. These tissues were cut into small pieces in tissue culture plates (Falcon, Becton Dickinson Labware, NJ) containing fresh media, and incubated at 37°C in fresh media for 24 hours, and supernatant fluid collected and stored at—20°C until analyzed. In another experiment, the terminal ileal tissue with the same preparation as described above were cultured with addition of 10% (v/v) either MRS or the conditioned medium derived from LF41, LGG, or BC41, at 37°C for 24 hours in tissue plates containing serum-free RPMI 1640 medium supplemented with P/S/F. The supernatants were collected and stored at—20°C until analyzed. PGE_2_ and TNF-α levels were analyzed by ELISA (R&D Systems), standardized to the tissue weight, and presented as the amount of cytokine per mg of tissue.

To assay the total hepatic PGE_2_, IL-10, or TNF-α protein concentration in the liver, the snap-frozen organs were homogenized in 1mL of PBS containing a protease inhibitor cocktail (Thermo Fisher Scientific, Rockford, IL Campus). The homogenates were centrifuged at 3,000 × *g* and 4°C for 12 min and stored at—20°C until analyzed. The levels of total protein in the supernatants were measured using a BCA Protein Assay Kit (Thermo Fisher Scientific). PGE_2_, TNF-α, or IL-10 concentrations in the supernatants were determined by ELISA kit (R&D Systems), standardized to the amount of total protein in supernatant, and presented as the amount of cytokine per mg of protein in supernatant.

### RNA Extraction and PCR

Total DNA was isolated and purified from diverse intestinal segments (terminal ileum, proximal colon, and terminal jejuna) using a QIAamp DNA spin column (Qiagen) according to the manufacturer’s recommended protocol. Total RNA from the intestinal segments or liver was isolated using an RNAeasy Miniprep Kit (Qiagen). Reverse transcription was performed using a GeneAmp RNA PCR kit (Applied Biosystems, MA). All samples were amplified using SYBR Green PCRmaster mix (Applied Biosystems) with primers specific toeither *Lactobacillus fermentum* 16S rRNA [21] or murine immune-associated mediators. Real-time quantitative PCR (q-PCR) was performed using a DNA Engine Opticon 2 apparatus (Bio-Rad, Hercules, CA) associated with the Opticon Monitor software (version 3.0, Bio-Rad). For quantification of *Lactobacillus fermentum* 16S rRNA gene copies in the intestinal tissues, plasmid standards and samples were assayed simultaneously. A range of concentrations from 10^1^ to 10^8^ of plasmid DNA was used in each real-time PCR assay to generate standard curves for quantitation of targeted bacterial 16S rRNA gene copies in test samples. Results are expressed as log_10_ of the 16S rRNA gene copies per mg of tissue samples. For quantification of each targeted mediator gene in the intestinal segments or liver, different MgCl_2_ (3 to 9mM) and primer concentrations (100 to 200 nM) were tested to optimize the PCR amplification. For all mediators, identical cycling conditions were used: initial step at 95°C for 3 min, followed by 38 to 43 cycles of denaturation at 94°C for 15 s, primer annealing at 60°C for 50 s, and extension at 72°C for 15 s. PCR products were then visualized by running on a 2% agarose gel with ethidium bromide. The relative expression of targeted mediator mRNA was normalized to that of the house-keeping β-actin mRNA in each sample, by using the 2^–△△CT^ cycle threshold method. Melt-curve analysis was used to confirm the authenticity of all PCR products. The primer sequences were synthesized by Sangon Biotech from Shanghai and shown in **S1 Table**.

To validate q-PCR for the quantitation of *Lactobacillus fermentum* (LF), 2 experiments were performed as follows. LF41, BC41, or LGG was cultured in MRS broth at 37°C overnight. An aliquot from each culture was dilution-plated on MRS agar (to enumerate each strain). Total bacterial genomic DNA was isolated from an aliquot of each culture and analyzed by q-PCR using primers specific to 16S rRNA of LF or LGG. To further evaluate the effectiveness of q-PCR quantification of LF from a mixed bacterial population, MRS broth was co-inoculated with both LGG and LF41 (low-, middle-, or high-dose), grown at 37°C overnight. Total bacterial genomic DNA was isolated from an aliquot of each of 3 samples and analyzed by q-PCR using primers specific to 16S rRNA of *Lactobacillus*, LF, or LGG, and each 16S rRNA gene copies were quantitated using the generated standard curves of plasmid standards as described in the previous paragraph. The ratio of the 16S rRNA gene copies determined by LF- or LGG-specific q-PCR to that by *Lactobacillus*-specific q-PCR was calculated, and added up. The primers specific for either *Lactobacillus* or LGG were used, and q-PCR conditions were performed, as previously reported [21–22].

### Western Blot

Proteins from terminal ileum were extracted with RIPA buffer after homogenization of tissues. Protein lysates were denatured and subjected to SDS-PAGE, and proteins were transferred to polyvinylidene difluoride membranes. The membranes were incubated with the primary Abs against COX-2, COX-1, and β-actin (Cell Signaling, Danvers, MA), followed by the appropriate species specific secondary horseradish peroxidase-conjugated antibodies (Cell Signaling, Danvers, MA). Detection was performed using an electrochemiluminescent detection system (Amersham, Buckinghamshire, UK).

### Antibody and Inhibitors Administration

To administer a neutralizing antibody specific to TNF-α (Anti-TNF), mice receiving either LF41 or PBS challenge were IP-injected every one day with Anti-TNF-α (0.2 mg/kg; R&D Systems) or its isotype IgG1 (0.2 mg/kg; R&D Systems), from day 1 to day 9.

To perform *in vivo* inhibition of activity of COX-2 or EP-4, mice orally receiving LF41 or PBS were given daily IP injection with a COX-2-specific inhibitor celecoxib (6 mg/kg; Sigma) or daily IG inoculation of a EP-4-specific inhibitor ONA-AE3-208 (I-EP4) (5 mg/kg; ApexBio, Boston, MA), from day 1 to day 10.

For administration of an IL-10-specific neutralizing antibody (Anti-IL-10) to evaluate its effect on LPS-induced serum ALT levels, mice pretreated with PBS or H-LF41 for 10 days were given IP injection with Anti-IL-10 (0.25 mg per mouse; BD Bioscience Pharmingen) or its isotype control IgG1 (0.25 mg per mouse; BD Bioscience Pharmingen) 30 minutes prior to LPS challenge.

In some experiments where mice were not inoculated with LPS, these mice were given IP inoculation of Anti-IL-10 (0.4 mg/kg) or its isotype control IgG1 (0.4 mg/kg) every one day, from day 1 to day 9.

ONO-AE3-208 (I-EP4) was dissolved in 0.003N NaOH, and celecoxib in 0.5% methylcellulose (Sigma). The dosage and vehicles for ONO-AE3-208 and celecoxib were guided by earlier reports [23–24].

### Myeloperoxidase (MPO) Activity

Frozen intestinal segments were assayed for MPO activity, as described previously [25].

### Bacterial Translocation and Intestinal Permeability

The bacterial titers in the liver and mesenteric lymph nodes were determined as our laboratory described [16]. Intestinal permeability was analyzed by oral administration of fluorescein isothiocyanate (FITC)-dextran (Sigma) at 400 mg/kg as previously described [26]. Before the administration, mice were fasted for 12 hours. Serum FITC-dextran concentrations were determined using an Envision 2104 Multiplate Reader (Perkin Elmer, Waltham, MA). Mice given 6 hours of high-dose LPS stimulation (10mg/kg BW; single IP injection) were used as the positive control.

### Statistical Analysis

An unpaired independent Student t-test was performed using a 95% confidence interval. One-way analysis of variance was used to analyze differences between groups, assisted by a post hoc Bonferroni test for multiple comparisons. All analyses were performed using GraphPad Prism (version 5.0). Differences were considered significant at P- values < 0.05.

## Results

### Oral pre-feeding mice with high-dose *Lactobacillus fermentum* ZYL0401 for 10 days alleviates LPS-induced expression of hepatic and serum TNF-α and liver injury

**Fig 1:**
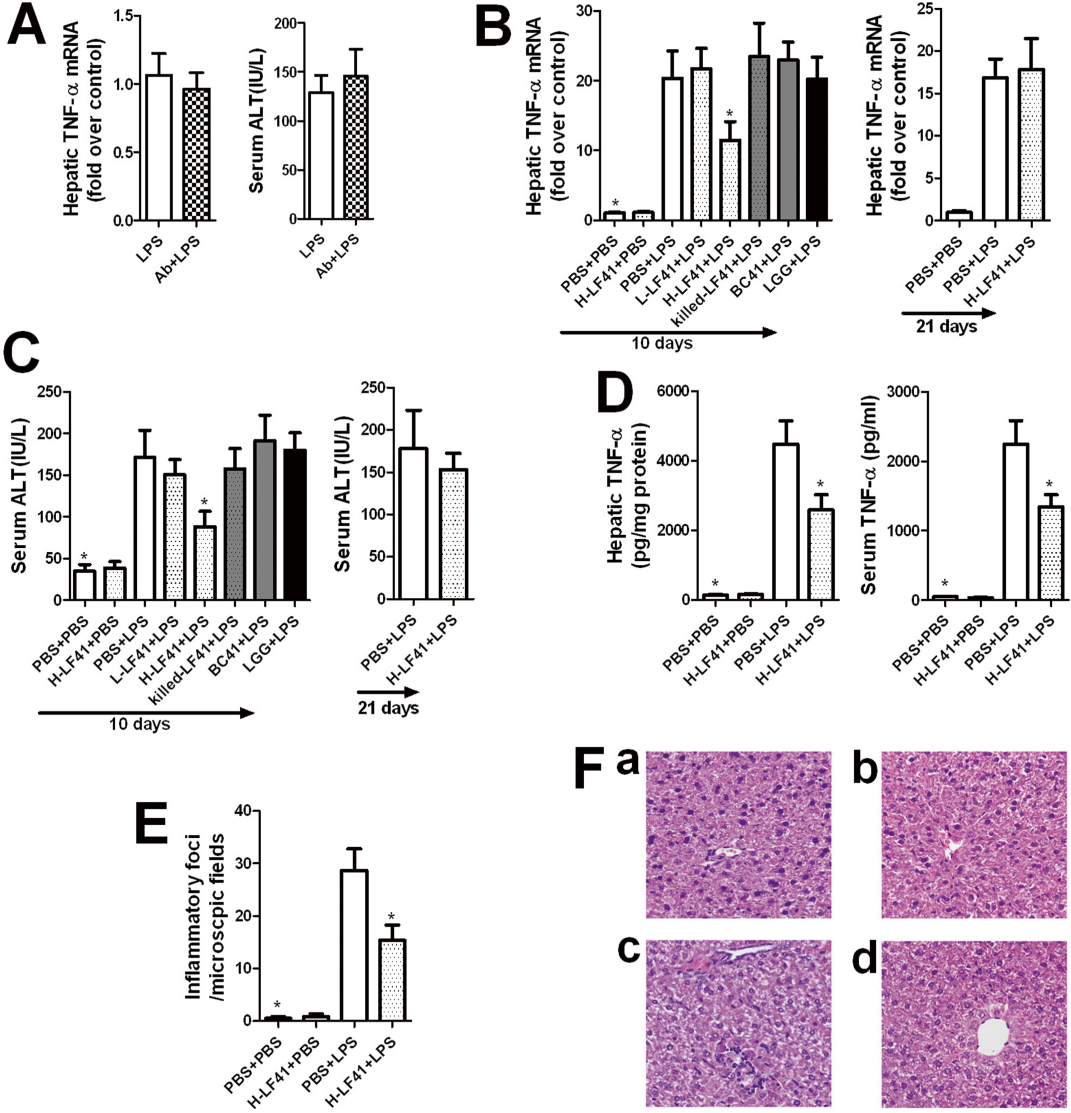
Orally-pretreated LF41 attenuates LPS-induced TNF-α expression and hepatic injury. **(A)** C57BL/6 mice (n = 8) either untreated or treated with antibiotic formula (Ab) were given single IP injection with LPS (500 μg/kg body weight). Mice were killed 2 and 16 h after LPS treatment for determination of hepatic TNF-α gene levels (left panel) by q-PCR and serum ALT activity (right panel), respectively. Results in the left panel are expressed as fold change relative to LPS. P > 0.05 compared to LPS. **(B)(C)** Mice (LPS-treated groups: n = 8–10 per group; the remainder: n = 6 per group) were given daily IG inoculation either for 10 consecutive days ofL-LF41, H-LF41, killed-LF41, LGG, BC41, or PBS, or for 21 consecutive days of either PBS or H-LF41 (right panel), and then single IP injection with LPS or PBS. Hepatic *Tnf* mRNA levels by q-PCR (B) and serum ALT activity (C) were determined. Results of (B) are expressed as fold change relative to PBS+PBS. H-LF41+LPS denotes 10 days of oral challenge with H-LF41 and then LPS injection, and other similar abbreviations conform to the same rule. * P < 0.05 compared to PBS+LPS. **(D)** Mice (LPS-treated groups: n = 8 per group; the remainder: n = 6 per group) were treated for 10 days with either PBS or H-LF41 and then challenged with LPS. Mice were killed 2 h after LPS treatment to test hepatic and serum TNF-α protein levels by ELISA.* P < 0.05 compared to PBS+LPS. **(E) (F)** Mice(LPS-treated groups: n = 12–14 per group; the remainder: n = 6–7 per group) pretreated for 10 days with PBS or H-LF were challenged with PBS or LPS. 20 h after the challenge, the inflammatory foci in the liver were determined (E), and representative histological outcomes of liver tissue were shown (F). * P < 0.05 compared to PBS+LPS. a: PBS+PBS; b: H-LF41+PBS; c: PBS+LPS; d: LF41+LPS. Values are shown as mean ± SEM. Results of **(A)** are representative of 2 experiments with similar results, and the remainder 3 experiments with similar results.

In a murine LPS-induced hepatic injury model (LPS dosage: 0.5 mg/kg; IP), we found that there was no increase in bacteria in the liver as well as mesenteric lymph nodes at 8, 16, or 24 hours after LPS treatment (**data not shown**). Furthermore, mice with an antibiotic formula pretreated to deplete intestinal commensal bacteria **(S1 Fig**) displayed no change in hepatic *Tnf* mRNA and serum ALT levels in this liver damage model compared with mice without receiving the antibiotics treatment **(Fig 1A**). Using this model we explored the preventive effect of several probiotic strains including LF41, LGG, and BC41 which have been shown to attenuate hepatic inflammation and liver injury in GalN-sensitized and alcoholic liver disease models [15–16, 18], on the TNF-α and ALT expression. We found that oral pretreatment of mice for 10 consecutive days with high-dose LF41 (H-LF41), but not low-dose LF41 (L-LF41) or high dose of LGG or BC41, resulted in attenuation of LPS-induced hepatic *Tnf* mRNA levels and serum ALT activity (**left panel of Fig 1B and 1C**). However, pretreatment with UV-killed H-LF41 had no similar effect as with H-LF41 (**left panel of Fig 1B and 1C**), indicating that the bacterial viability is indispensable to the preventive capacity. The duration of pretreatment was also crucial to determination of the outcomes, as evidenced by no alteration in either the hepatic *Tnf* mRNA or serum ALT levels after 3 weeks of pretreatment with H-LF41 (**right panel of Fig 1B and 1C**). Expectedly, pretreatment with H-LF41 for 10 days showed pronounced attenuation of LPS-induced hepatic and serum TNF-α protein levels (**Fig 1D**). Moreover, histological analysis showed that 10 days of pretreatment with H-LF41 significantly reduced infiltration of inflammatory cells into the liver in response to LPS challenge (**Fig 1E and 1F**).

### Administration of H-LF41 for 10 days prominently enhances the amount of DNA specific to *Lactobacillus fermentum* in the ileum

**Fig 2:**
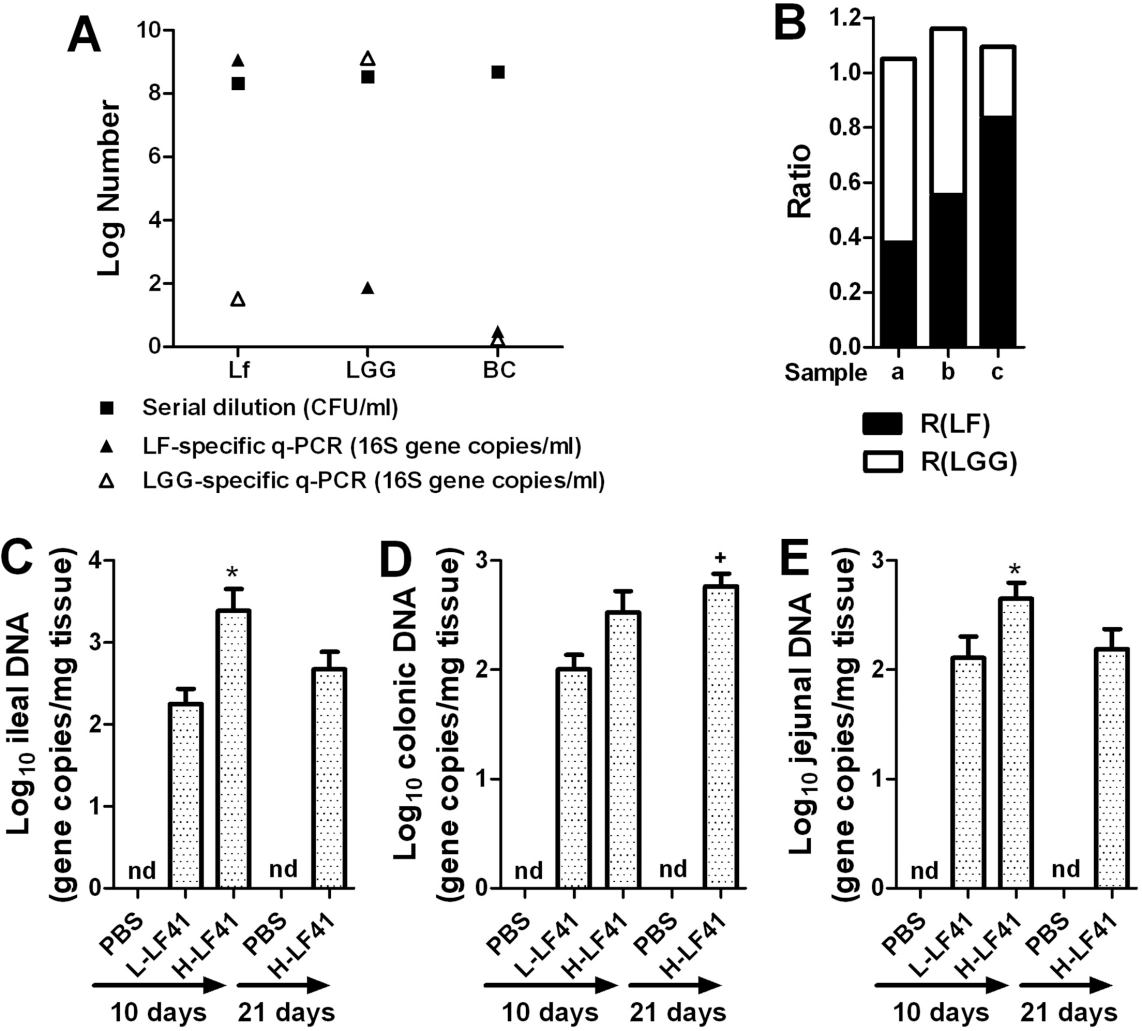
Validation of q-PCR for quantitation of LF and effect of LF41 administration on LF-specific 16S rRNA levels in intestinal tissues. **(A)** LF41, BC41, or LGG was cultured in MRS broth at 37°C overnight. An aliquot of culture from each culture was dilution-plated on MRS agar (to enumerate each strain). Total bacterial genomic DNA was isolated from an aliquot of each culture and analyzed by q-PCR using the primers specific to the 16S rRNA of either LF or LGG. Black and white triangles denote log numbers of 16S rRNA gene copies determined by LF- and LGG-specific q-PCR, respectively; black squares denote log numbers of bacteria determined by serial dilution. **(B)** MRS broth was co-inoculated with LGG and low, middle, or high dose of LF41, grown at 37°C overnight. Total bacterial genomic DNA was isolated from an aliquot of each sample and analyzed by q-PCR using the primers specific to 16S rRNA of *Lactobacillus*, LF, or LGG. The samples of a, b, and c denote the co-cultures of LGG with low, middle, and high dose of LF41, respectively; “R(LF)” and “R(LGG)” denote the ratios of the respective 16S rRNA gene copies determined by LF- and LGG-specific q-PCR to the gene copies by *Lactobacillus*-specific q-PCR. **(B)(C)(D)** Mice (n = 8) were orally inoculated either for 10 days with PBS, L-LF41, or H-LF41, or for 3 weeks with PBS or H-LF41, and LF-specific 16S rRNA gene levels in terminal ileum **(B)**, proximal colon **(C)**, and distal jejuna **(D)** determined by q-PCR. Results are expressed as log_10_ of the 16S rRNA gene copies per mg of tissue samples. Values of are shown as mean ± SEM. * P < 0.05 compared to L-LF41 or H-LF41 (21 days); **+** P < 0.05 compared to H-LF41 (10 days); nd, not detected. Results are representative of 2 experiments with similar results.

To explore the murine host responses after LF41 treatment, we firstly turned to the analysis of intestinal microbial alteration after LF41 administration via q-PCR of the amount of *16S rRNA* gene specific to *Lactobacillus fermentum*(LF) in diverse intestinal tissues. To validate the use of q-PCR for quantification of LF [21], adjuvant q-PCR for quantification of LGG was also performed [22]. It was shown that the gene copy numbers of LF or LGG determined by q-PCR was well closed to the bacterial number of either enumerated via serial dilution; however, the numbers determined by either specific q-PCR of the remaining bacteria were much fewer than that by serial dilution (**Fig 2A**). Thus, the favorable specificity and sensitivity of both LF- and LGG-specific q-PCR was validated. To examine the effectiveness of using q-PCR for quantification of LF in a bacterial mixture, MRS broth was co-inoculated with LGG and LF41 in a series of increasing doses, grown overnight. The gene copies of an aliquot of each were determined by q-PCR specific to *Lactobacillus*, LF, or LGG. The ratio of the gene copies determined by either LF- or LGG-specific q-PCR to the gene copies by *Lactobacillus*-specific q-PCR was calculated (denoted as R(LF) or R(LGG)), and these two ratios of each sample were added up. It was shown that the sum of the ratios of each sample was approximately 1 and R(LF)s from three samples were correlated well (**Fig 2B**).

After administration of H-LF41 for 10 days, the amount of LF-specific DNA was prominently higher in the terminal ileum than in the terminal jejuna or proximal colon (**Fig 2C, 2D and 2E**). Importantly, mice that underwent 10 days challenge of H-LF41 produced much higher levels of this DNA in the terminal ileum than did mice fed either L-LF41 for 10 days or H-LF41 for 3 weeks (**Fig 2C**). In contrast, mice administered H-LF41 for 3 weeks showed a higher amount in the proximal colon compared with mice administered H-LF41 for 10 days (**Fig 2D**).

### Ten days of H-LF41 challenge significantly enhanced expression of COX-2 and IL-10 in the ileum

**Fig 3:**
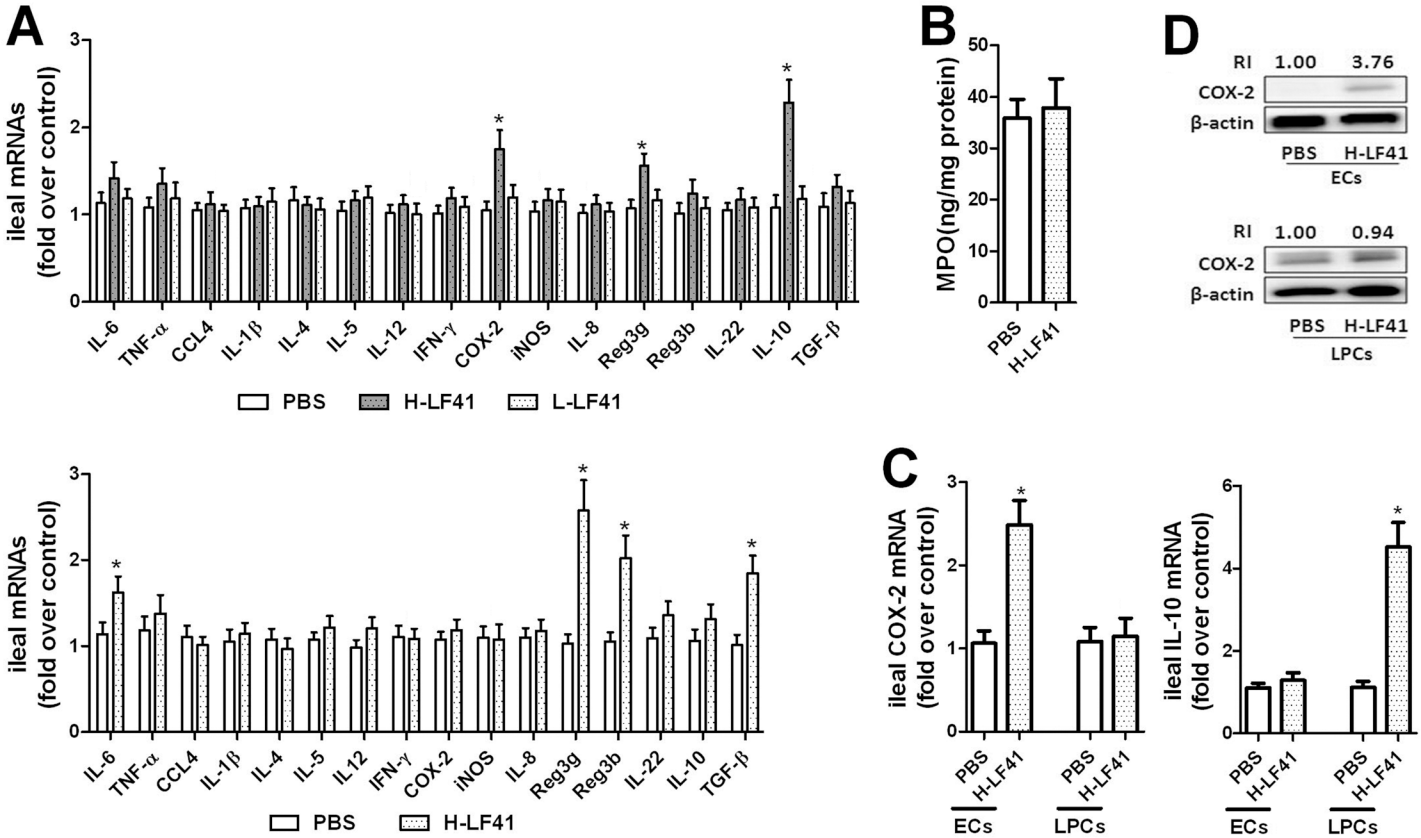
Ten days of H-LF41 treatment significantly enhances ileal expression of COX-2 and IL-10. **(A)** q-PCR for mRNA levels of several factors associated with innate and adaptive immune responses in the terminal ileum collected from mice (n = 10) fed either for 10 days with PBS, L-LF41, or H-LF41 (upper panel), or for 3 weeks with either PBS or H-LF41 (lower panel). Results are expressed as fold change relative to “PBS”. * P < 0.05 compared to PBS. **(B)** MPO expression in the terminal ileum from mice (n = 6) treated with either PBS or H-LF41 for 10 days. P > 0.05 compared to PBS. **(C)** Epithelial cells (ECs) from the terminal ileum and its underlying lamina propria cells (LPCs) were isolated from mice (n = 8) orally given10 days supplement of PBS or H-LF41. *Cox2* and *Il10* mRNA levels in these cells were determined by q-PCR. Results are expressed as fold change relative to PBS. * P < 0.05compared to PBS. **(D)** Western blot assay for representative COX-2 protein levels in ECs and LPCs of the terminal ileum of mice (n = 4) fed either PBS or H-LF41 for 10 days. “RI” denotes the mean relative luminous intensity of the targeted protein band, which is positively correlated with the real luminance; the RI in the control group is set at 1.00. All values except that of Western blot are shown as mean ± SEM. Results are representative of 2 similar experiments.

After oral treatment of LF41, we evaluated gene levels of some immune-associated factors in the terminal ileum. These included some pro- and anti-inflammatory mediators, two antimicrobial peptides REG3γ and REG3β with their high expression observed in the epithelial cells and paneth cells of the ileum [27], and several cytokines related to innate and adaptive immune responses. It was shown that 10 days administration of H-LF41 pronouncedly increased mRNA levels of *Cox2*, *Il10*, and *Reg3g* in the terminal ileum compared with the control mice, whereas treatment of L-LF41 for 10 days had no obvious effect on the levels of these factors (**upper panel of Fig 3A**). In contrast, mice challenged with H-LF41 for 3 weeks showed no alteration in either *Cox2* or *Il10* mRNA levels but pronounced increase in the levels of *Reg3g*, *Reg3b*, *Tgfb1*, and *Il6* in the terminal ileum (**lower panel of Fig 3A**). In addition, none of *Cox2*, *Il10*, and *Reg3g* levels were significantly altered in either the terminal jejuna or proximal colon after feeding H-LF41 for 10 days (**S2 Fig**). Although showing increased expression of COX-2 with pro-inflammatory capability [28], mice given 10 days of H-LF41 did not show abnormalities, such as bleeding, swelling, or edema, in the ileum (**data not shown**). Consistently, these mice also did not show a significant change in ileal MPO levels compared with the control mice (**Fig 3B**). Furthermore, in the terminal ileum of mice fed H-LF41 for 10 days, upregulation of *Cox2* was found to be limited to the epithelial cells but not the underlying lamina propria cells, whereas *Il10* levels were prominently enhanced in the lamina propria cells but not epithelial cells (**Fig 3C**). Consistent with the distribution of *Cox2* gene, increased COX-2 protein was also observed to be restricted in the epithelial cells but not lamina propria cells after H-LF41 administration (**Fig 3D**).

### Ten days of H-LF41 administration increases hepatic PGE_2_ without affecting either hepatic COX-1 or COX-2 expression; PGE_2_-EP4 pathway is responsible for the inhibition of hepatic TNF-α expression

**Fig 4:**
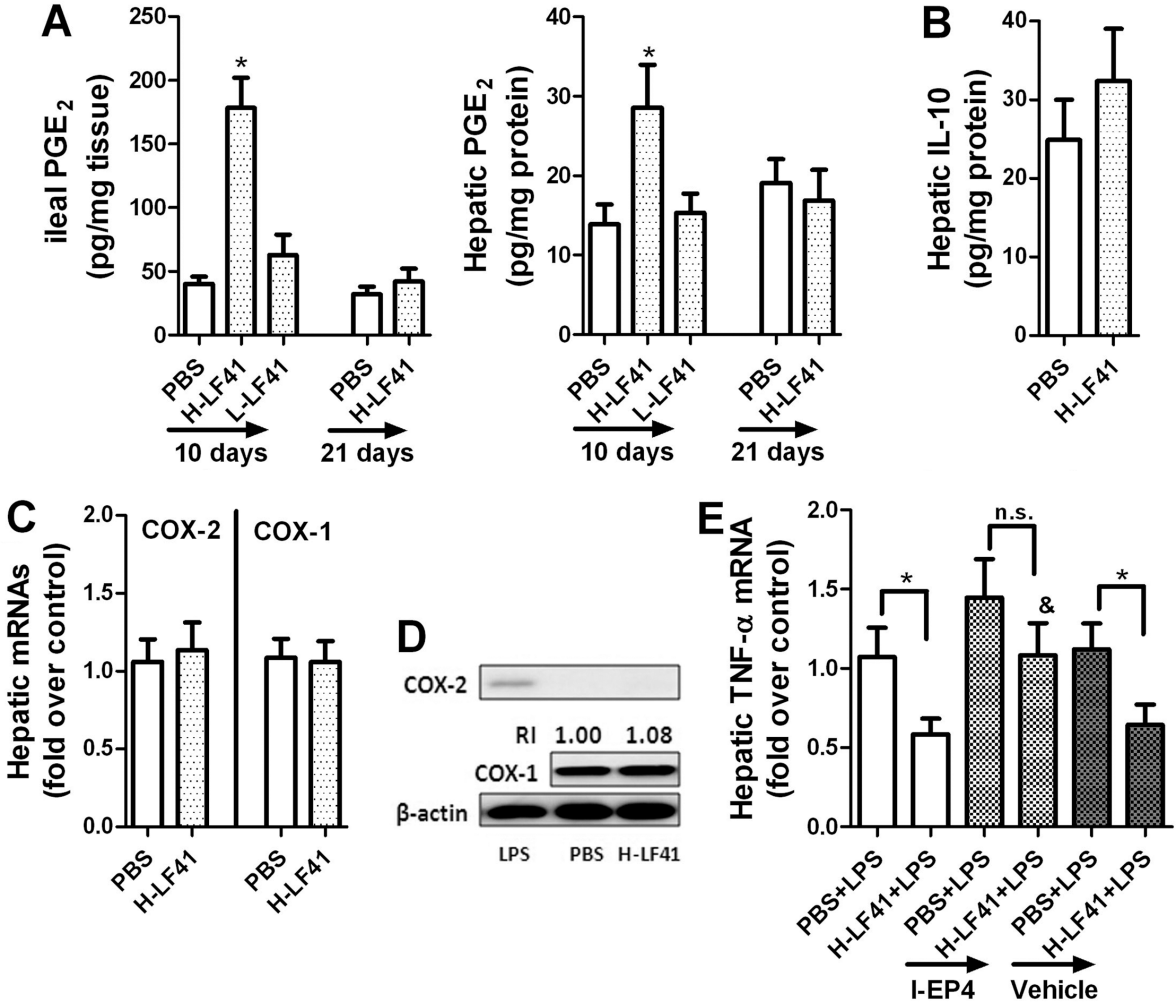
PGE_2_-EP4 pathway is in charge of LF41-mediated attenuation of hepatic TNF-α expression. **(A)** ELISA for PGE_2_ secretion by the terminal ileum and total PGE_2_ levels in the liver of mice (n = 8) orally treated either for 10 days with PBS, L-LF41, or H-LF41, or for 3 weeks with PBS or H-LF41. * P < 0.05 PBS. **(B)** ELISA for hepatic IL-10 protein concentration of mice (n = 8) fed either PBS or H-LF41 for 10 days. P > 0.05 compared to PBS. **(C)** q-PCR for hepatic *Cox1* or *Cox2* mRNA levels of mice (n = 8) fed either PBS or H-LF41 for 10 days. Results are expressed as fold change relative to PBS. P > 0.05 compared to PBS. **(D)** Western blot assay for representative hepatic COX-1 and COX-2 protein levels of mice (n = 4) orally treated with either PBS or H-LF41 for 10 days. Hepatic COX-2 protein levels from a mouse receiving 2 h of stimulation with LPS (0.5 mg/kg BW; single IP injection) were determined as a positive control (left lane). **(E)** Mice (H-LF41-treated groups: n = 10 per group; PBS-treated groups: n = 8 per group) were pretreated with 10 days of PBS or H-LF41, either alone or combined with administration of either a specific inhibitor for PGE_2_ receptor EP-4, ONA-AE3-208 (I-EP4), or its vehicle (Vehicle). Hepatic *Tnf* mRNA levels were assayed by q-PCR 2 h after LPS treatment. Results are expressed as fold change relative to PBS+LPS.* P < 0.05; **&** P < 0.05 compared to H-LF41+LPS; **n.s.**, non-statistical difference. All values except that of Western blot are shown as mean ± SEM. Results are representative of 2 similar experiments.

Given the close vascular and lymphatic relationship between the liver and intestine, we also examined whether hepatic expression of IL-10, COX-2, or PGE_2_ could be altered after challenge with H-LF41 for 10 days. Indeed, these mice exhibited remarkable upregulation of not only ileal PGE_2_ secretion but also hepatic PGE_2_ amount compared with the control mice (**Fig 4A**). However, treatment with either L-LF41 for 10 days or H-LF41 for 3 weeks had no similar effect as with H-LF41 for 10 days (**Fig 4A**). In addition, administration of either LGG or BC41 for 10 days showed insignificant regulatory effect on the total hepatic PGE_2_ levels (18.1 **±** 3.2 in PBS-treated mice; 21.2 **±** 4.7 in LGG-treated mice; 15.7 **±** 4.0 in BC41-treated mice). There was also no alteration in total hepatic IL-10 protein levels after 10 days supplement of H-LF41 (**Fig 4B**). Although displaying increased hepatic PGE_2_ levels, strikingly, LF41-treated mice did not exhibit significant alteration in either hepatic *Cox1* or *Cox2* gene levels in comparison with the control mice (**Fig 4C**), nor was a significant change in protein amount of either (**Fig 4D**). It should be pointed out that hepatic COX-2 protein was not detected in either PBS- or LF41-treated mice (**Fig 4D**).

PGE_2_ has been shown to inhibit LPS-activated serum TNF-α production [29]. PGE_2_-EP4 pathway has been demonstrated to be mainly responsible for *in vitro* inhibition by PGE_2_ of LPS-induced TNF-α expression in Kupffer cells [30]. To evaluate whether this pathway was involved in the prevention of LPS-stimulated hepatic *Tnf* gene levels, mice were pre-fed for 10 days with PBS or H-LF41 singly or in combination with administration of an EP4-specific inhibitor, ONO-AE3-208 (I-EP4), or its vehicle, and the hepatic *Tnf* mRNA levels in these mice determined after LPS challenge. Indeed, the EP4-specific blockade resulted in abrogation of LF41-mediated attenuation of hepatic *Tnf* mRNA levels (**Fig 4E**). Furthermore, administration of PGE_2_ 3 hours before LPS challenge also significantly attenuated hepatic *Tnf* mRNA levels (**S3 Fig**).

### COX-2 and IL-10 blockade eliminates and facilitates LF41-mediated upregulation of hepatic PGE_2_ levels, respectively

**Fig 5:**
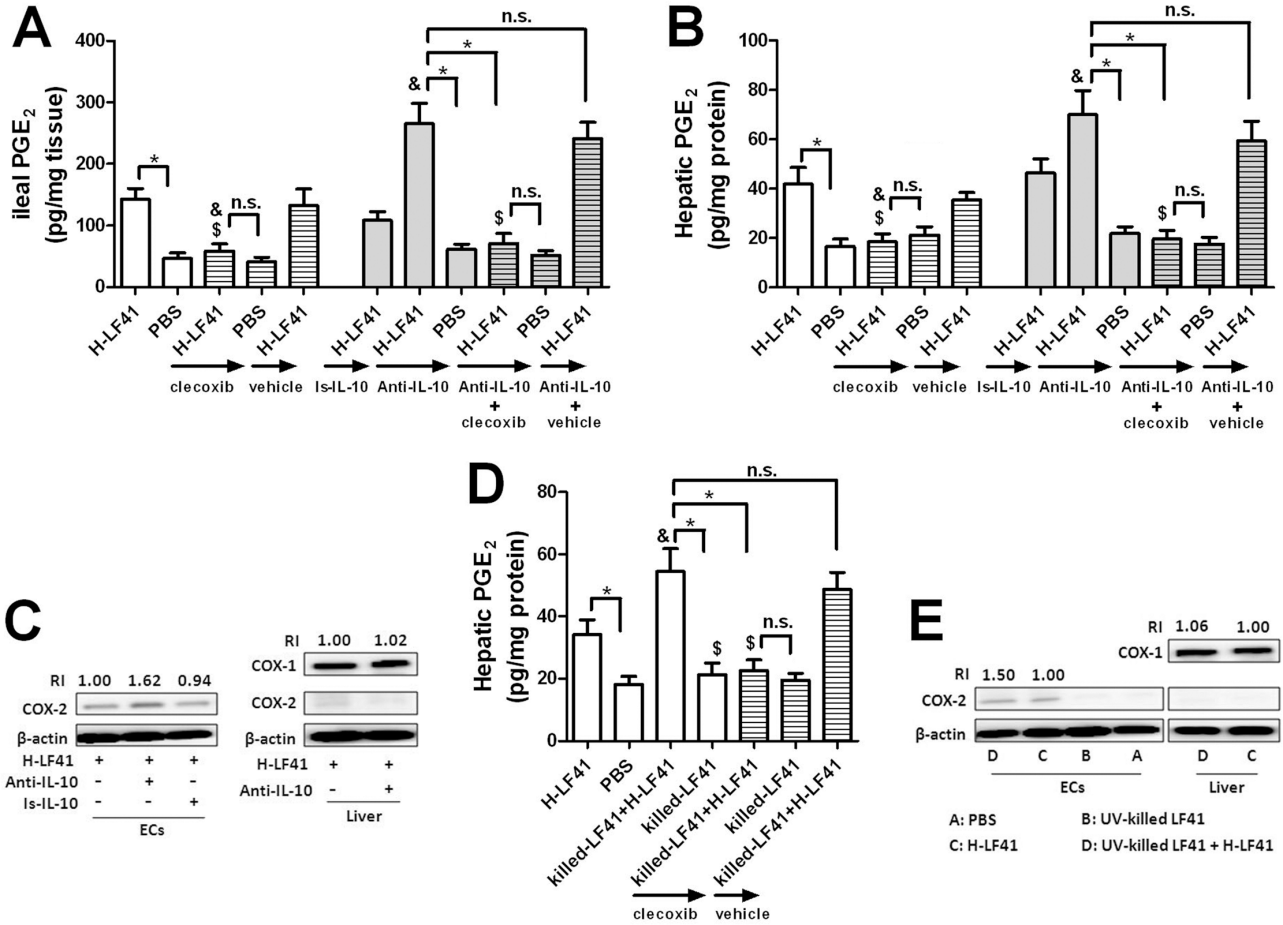
LF41-mediated upregulation of PGE_2_ is abrogated by COX-2 blockade, but facilitated by IL-10 blockade in a COX-2-dependent manner. **(A)(B)** ELISA for PGE_2_ secretion by the terminal ileum (A) and PGE_2_ amount in the liver (B) of mice (PBS-treated groups: n = 5–6 per group; H-LF41-treated groups: n = 8 per group) treated with a combination of either PBS or H-LF41 for 10 days, either alone or in combination with the COX-2-specific inhibitor celecoxib, its vehicle (vehicle), IL-10-specific antibody (Anti-IL-10), its isotype control (Is-IL-10), or Anti-IL-10 together with either celecoxib or its vehicle. * P < 0.05; **&** P < 0.05 compared to H-LF41; **$** P > 0.05 compared to PBS; **n.s.**, non-statistical difference. **(C)(E)** Western blot assay for representative protein levels of COX-2 in epithelial cells (ECs) of the terminal ileum and COX-2 and COX-1 in the liver from mice (n = 4) either given 10 days H-LF41 treatment together with IL-10 blockade (C), or treated with 10 days of PBS, killed-LF41, H-LF41, or killed-LF41 together with H-LF41 (killed-LF41+H-LF41) (E). **(D)** ELISA for hepatic PGE_2_ amount in mice (n = 8) fed PBS or H-LF41 for 10 days or given a combination of 10 days gavage of killed-LF41 or killed-LF41+H-LF41,either singly or combined with COX-2 blockade. * P < 0.05; **&** P < 0.05 compared to H-LF41; **$** P > 0.05 compared to PBS. All values except that of Western blot are shown as mean ± SEM. Results are representative of 2 similar experiments.

Due to the significance of the upregulated hepatic PGE_2_, we explored the mechanism by which H-LF41 administration increased hepatic PGE_2_ but without modulating either hepatic COX-1 or COX-2 expression. Considering the close anatomical relationship between the liver and intestine as well as the enhanced COX-2 expression and PGE_2_ secretion in the terminal ileum of LF41-fed mice, we hypothesized that a specific blockade of COX-2 activity via treatment of a COX-2-specific inhibitor, celecoxib, might eliminate LF41-elicited enhancement of hepatic PGE_2_ level. Indeed, the COX-2 blockade terminated the upregulation of not only ileal PGE_2_ secretion but also total hepatic PGE_2_ levels in LF41-treated mice (**Fig 5A and 5B**).

Because of the enhanced IL-10 expression in the terminal ileum after 10 days of H-LF41 gavage (**Fig 3C**), we also examined whether antibody-mediated specific blockade of IL-10 could have a similar action as that of COX-2 blockade. Contrary to results of the COX-2 blockade, the IL-10 blockade brought about further increase in the already upregulated hepatic and ileal PGE_2_ levels associated with LF41 treatment (**Fig 5A and 5B**). This stimulatory effect was also controlled by COX-2 in LF41-fed mice, as evidenced by dramatically lower levels of both hepatic and ileal PGE_2_ after H-LF41 treatment in combination with both IL-10 and COX-2 blockades than after H-LF41 treatment in combination with the IL-10 blockade (**Fig 5A and 5B**). Moreover, PGE_2_ levels of either in mice treated with H-LF41 in combination with both IL-10 and COX-2 blockades was not significantly different from that of the PBS treatment (**Fig 5A and 5B**). Furthermore, in LF41-fed mice, the IL-10 blockade promoted COX-2 protein levels in the epithelial cells of the terminal ileum, whereas the hepatic COX-2 protein was not detected and the COX-1 protein levels not affected after the blockade (**Fig 5C**).

Interestingly, mice co-administered H-LF41 and killed LF41 for 10 days also had higher levels of hepatic PGE_2_ than did mice treated with H-LF41 alone, and the increased levels were diminished after the COX-2 blockade (**Fig 5D**). However, challenge with killed LF41 alone showed no effect on hepatic PGE_2_ levels compared with the PBS treatment (**Fig 5D**). The COX-2 protein levels in the epithelial cells of the terminal ileum were significantly enhanced after the co-administration compared with that after H-LF41 administration, whereas treatment with killed LF41 alone showed no effect (**Fig 5E**). Similar to the IL-10 blockade, the co-administration also did not affect protein levels of hepatic COX-2 and COX-1 (**Fig 5E**).

### H-LF41 pretreatment augments LPS-activated hepatic IL-10 levels in a COX-2-dependent manner, causing the attenuation of serum ALT

**Fig 6:**
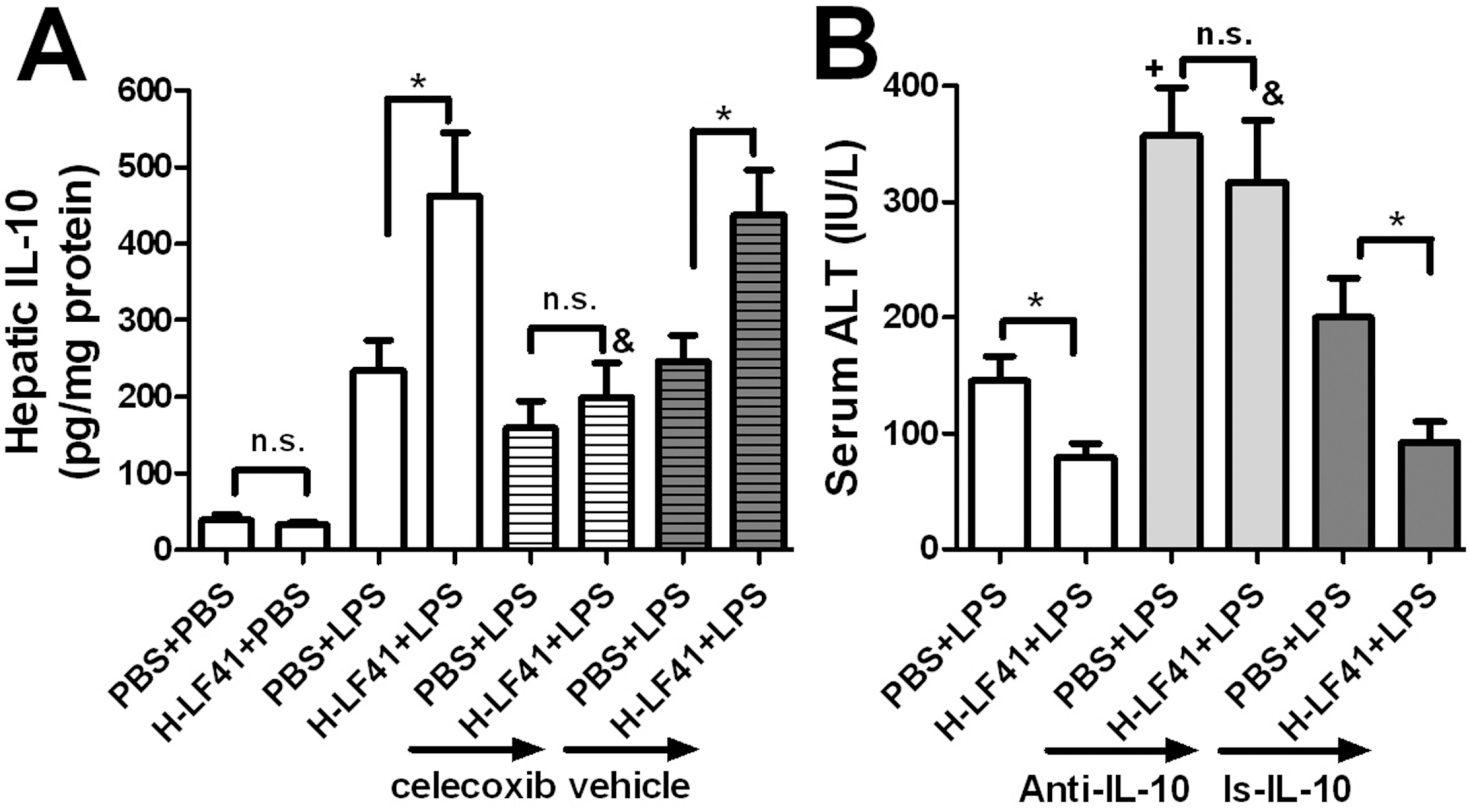
LF41 pretreatment significantly increased LPS-activated hepatic IL-10, which accounts for the attenuation of serum ALT levels. **(A)** Mice (LPS-treated groups: n = 8–10 per group; the remainder: n = 6 per group) were pretreated PBS or H-LF41 for 10 days, either alone or in combination with celecoxib or its vehicle, and then given injection of either PBS or LPS. Four hours after LPS challenge, mice were killed to test hepatic IL-10 protein levels by ELISA.* P < 0.05; **&** P < 0.05 compared to H-LF41+LPS; **n.s.**, non-statistical difference. **(B)** Mice (H-LF41-treated groups: n = 10 per group; PBS-treated groups: n = 8 per group) were pretreated with PBS or H-LF41 for 10 days, either singly or in combination with IL-10-specific antibody (Anti-IL-10) or its isotype control (Is-IL-10) 30 min prior to LPS inoculation. Serum ALT levels were determined 16 h after LPS treatment. * P < 0.05; **+** P < 0.05 compared to PBS+LPS; **&** P < 0.05 compared to H-LF41+LPS. Values are shown as mean ± SEM. Results are representative of 2 experiments with similar results.

PGE_2_ augments LPS-activated IL-10 expression in macrophages *in vitro* [29]. Consistently, we also demonstrated that H-LF41 pretreatment for 10 days was associated with aggrandizement of LPS-induced hepatic IL-10 protein, while LF41 itself had no stimulatory effect on hepatic IL-10 levels in the absence of LPS (**Fig 6A**). This amplification effect on hepatic IL-10 levels was abolished after COX-2 blockade (**Fig 6A**). Furthermore, LF41-mediated alleviation of serum ALT activity induced by LPS was abrogated by administration of IL-10-specific antibody 30 minutes prior to LPS treatment (**Fig 6B**). Since the isotype control for the antibody had no similar effect (**Fig 6B**), the inhibition of serum ALT expression was indeed dependent on the IL-10. In addition, pretreatment of PGE_2_ also significantly facilitated hepatic *Il10* mRNA levels and reduced serum ALT levels in response to LPS challenge (**S3 Fig**).

### COX-2 in LF41-fed mice prevents enhancing TNF-α expression in the liver and terminal ileum and averts TNF-α-mediated enhancement of intestinal permeability

**Fig 7:**
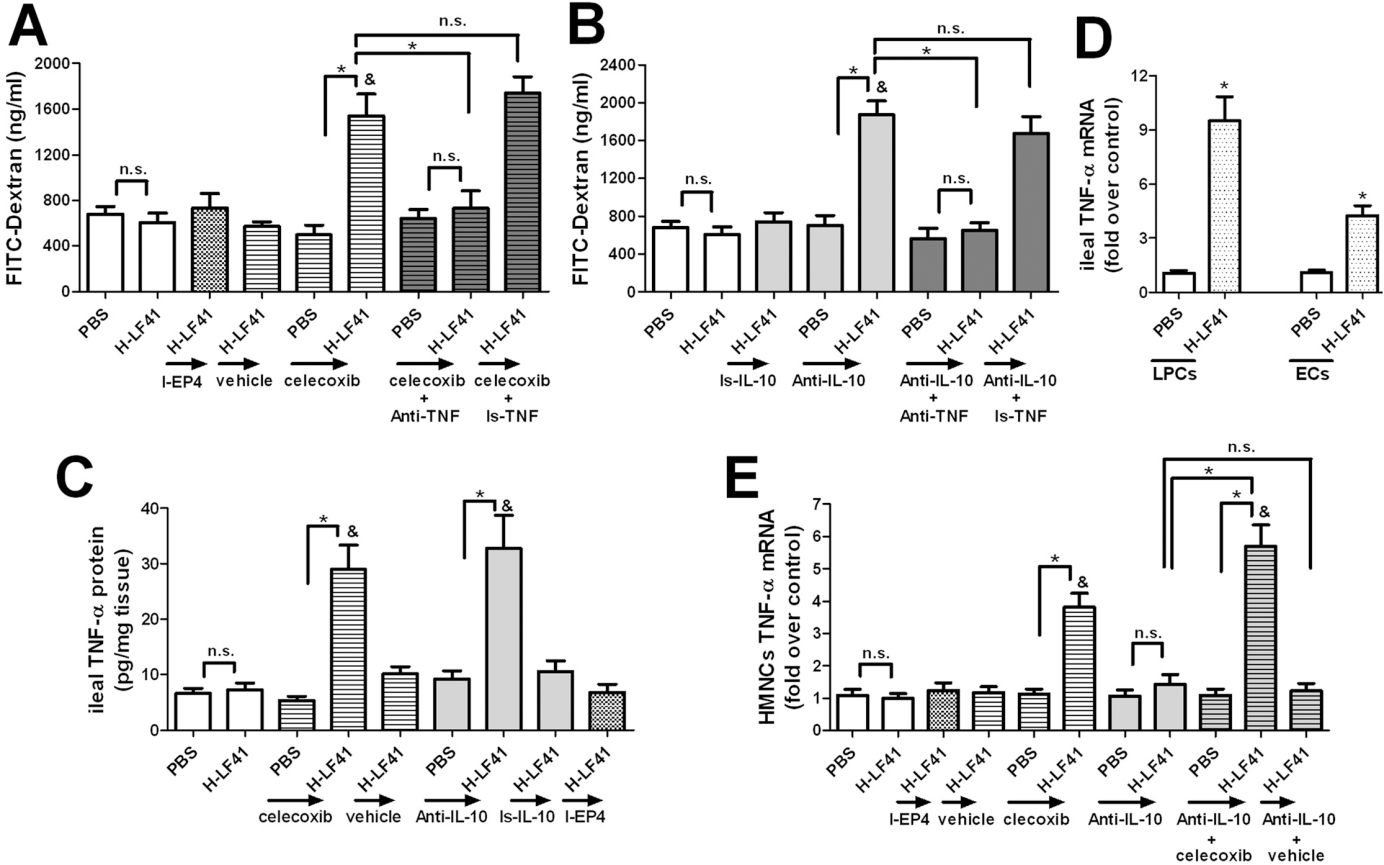
Effect of COX-2 or IL-10 blockade on TNF-α expression and intestinal permeability in LF41-fed mice. **(A)(B)** Mice (PBS-treated groups: n = 5 per group; H-LF41-treated groups: n = 7 per group) were given 10 days treatment of PBS or H-LF41, either alone or in combination with blockade of EP4, COX-2, or IL-10, or co-blockade of TNF-α with COX-2 or IL-10, and then given IG inoculation with FITC-Dextran. Three hours later, the FITC-Dextran amount in the blood was determined. I-EP4, EP4-specific inhibitor; vehicle, the vehicle for celecoxib; celecoxib, COX-2-specific inhibitor; Anti-TNF, TNF-α-specific antibody; Is-TNF, the isotype control for Anti-TNF; Anti-IL-10, IL-10-specific antibody; Is-IL-10, the isotype control for Anti-IL-10.* P < 0.05; & P < 0.05compared to H-LF41; **n.s.**, non-statistical difference. **(C)** ELISA for TNF-α secretion by the terminal ileum collected from mice (PBS-treated groups: n = 5 per group; H-LF41-treated groups: n = 7 per group) fed for 10 days PBS or H-LF41, either singly or in combination with blockade of COX-2, IL-10, or EP4. * P < 0.05; & P < 0.05 compared toH-LF41. **(D)** q-PCR for *Tnf* mRNA levels in the epithelial cells (ECs) and lamina propria cells (LPCs) of the terminal ileum from mice (n = 7) treated for 10 days with PBS or H-LF41 in the presence of celecoxib administration. Results are expressed as fold change relative to PBS. * P < 0.05. **(E)** q-PCR for *Tnf* mRNA levels in the HMNCs isolated from mice (PBS-treated groups: n = 6 per group; H-LF41-treated groups: n = 8–10 per group) given 10 days treatment of PBS or H-LF41, either alone or together with blockade of EP4, COX-2, or IL-10, or with co-blockade of IL-10 and COX-2. Results are expressed as fold change relative to PBS. * P < 0.05; **&** P < 0.05 compared to H-LF41. Values are shown as mean ± SEM. Results are representative of 2 similar experiments.

Having observed the pivotal role of COX-2 in modulating the upregulation of hepatic PGE_2_ levels associated with LF41, we investigated the potential other function of COX-2 in LF41-treated mice. Although there was no significant change in intestinal epithelial permeability after oral treatment of H-LF41 for 10 days (**Fig 7A**), pronounced increase in intestinal permeability was found in LF41-treated mice after blockade of COX-2, but not PGE_2_ receptor EP4 (**Fig 7A**). There was no apparent alteration after H-LF41 gavage for 10 days in the expression of pro-inflammatory cytokines (**Fig 3A**), such as TNF-α, IFN-γ, and IL-1β that can impair intestinal permeability *in vitro* and *in vivo* [31]. Therefore, we examined whether the COX-2 blockade would influence the levels of these pro-inflammatory cytokines in LF41-fed mice. Indeed, these mice exhibited enhanced TNF-α expression after the blockade, with increased TNF-α secretion in the terminal ileum and upregulated *Tn f*mRNA levels in both the epithelial cells and lamina propria cells of the terminal ileum (**Fig 7C and 7D**). However, the mRNA levels of *Ifng* and *Il1b* in the terminal ileum was not affected by the blockade (**data not shown**). Moreover, exposure of isolated terminal ileal tissue to LF41-derived conditioned medium also markedly increased TNF-α secretion by the tissue (**S4 Fig**). This implied that LF41 possesses the capacity to induce TNF-α expression in intestinal tissues if in direct contact with them.

We hypothesized that the upregulated TNF-α might regulate the enhancement of intestinal permeability that was observed after the COX-2 blockade. To this end, we examined the influence of the COX-2 blockade with or without the TNF-α blockade on intestinal permeability in LF41-fed mice. It was shown that co-administration of celecoxib and TNF-α-specific antibody abrogated celecoxib-mediated increase in intestinal permeability (**Fig 7A**). This indicated that in LF41-challenged mice, COX-2 was required for preventing intestinal permeability from being enhanced by TNF-α. In view of the upregulated IL-10 in the ileum of LF41-fed mice, we also examined whether the IL-10 blockade would have the same effect as COX-2 blockade. Indeed, IL-10 blockade also led to an increase in ileal TNF-α secretion (**Fig 7C**). Moreover, IL-10 blockade exerted an effect on intestinal permeability that was similar to that seen with the COX-2 blockade, and the upregulated TNF-α was also responsible for the increased intestinal permeability after the IL-10 blockade (**Fig 7B**). Consistent with its failure to increase intestinal permeability, inhibition of EP4 activity also had no influence on ileal TNF-α secretion in LF41-fed mice (**Fig 7C**).

Based on the enhanced intestinal permeability and ileal TNF-α production after either COX-2 or IL-10 blockade, we hypothesized that the COX-2, IL-10, or both in LF41-challenged mice might also be required for preventing enhancement of hepatic TNF-α expression. To test this, HMNCs were isolated from mice after 10 days administration of either PBS or H-LF41, singly or together with blockade of COX-2 or IL-10 or with a co-blockade of IL-10 and COX-2, and then *Tnf* mRNA levels in the HMNCs were determined. The mRNA levels of *Tnf* in mice treated with only H-LF41 were similar to those of the PBS-fed mice (**Fig 7E**). In addition, mice treated with H-LF41 in combination with either EP4 or IL-10 blockade showed no alteration in this mRNA levels compared with the LF41-fed mice. However, the levels were pronouncedly upregulated in LF41-fed mice after either COX-2 blockade or co-blockade of COX-2 and IL-10 (**Fig 7E**).

## Discussion

The interaction between probiotics and the gut is well documented, and the direct modification on intestinal function by orally-administered probiotics accounts for a large part of their biological effect. These include maintenance of intestinal microbial homeostasis, protection of gut barrier function, and influence on innate and adaptive immunity in the intestine [10]. The liver has a close anatomical relationship with the gut. However, little is known about whether oral administration of probiotics could also directly affect the liver, as in the intestine. In this study, we demonstrated that in a liver injury model induced by IP injection of low-dose LPS, hepatic injury and inflammation may be largely unaffected by intestine-derived microbial components. Using this model, we found that oral pretreatment with H-LF41 attenuated hepatic injury and inflammation, with significantly reduced serum ALT levels, infiltration of inflammatory cells into the liver, and hepatic and serum TNF-α expression. Furthermore, the up-regulation of hepatic PGE_2_ was observed after H-LF41 challenge, which was not accompanied with enhancement of either hepatic COX-2 or COX-1 expression. Similarly, H-LF41 pretreatment also significantly increased hepatic IL-10 levels in the presence of LPS. Importantly, PGE_2_-EP4 pathway and IL-10 in H-LF41-pretreated mice were responsible for the inhibition of LPS-induced hepatic *Tnf* mRNA and serum ALT levels, respectively. In H-LF41-pretreated mice, the enhancement of hepatic PGE_2_ and IL-10 levels were regulated by COX-2, the expression of which was significantly enhanced in the ileum but not liver. Furthermore, COX-2 in H-LF41-pretreated mice prevented enhancing *Tnf* mRNA levels in the liver and terminal ileum and avoided TNF-α-mediated increase in intestinal permeability.

The dosage of LF41, dose and duration of treatment, is crucial to the outcomes in our model. Interestingly, this strain seems to have a predilection for the ileum in mice after oral administration for 10 days of either H-LF41 or L-LF41, as the amount of DNA specific to LF in the terminal jejuna or proximal colon was remarkably lower than that in the terminal ileum. Importantly, after LF41 challenge for 10 days, the dose of LF41 positively correlated with ileal amount of the DNA, ileal expression of COX-2, IL-10, or PGE_2_, hepatic PGE_2_ levels, or the inhibition of hepatic *Tnf* mRNA or serum ALT levels. However, 21 days of administration of H-LF41 had lower levels of LF-specific DNA in the terminal ileum than did 10 days of H-LF41 treatment. Besides, there was no alteration in the levels of hepatic PGE_2_ or ileal COX-2 after 21 days treatment with H-LF41. Consistently, mice fed H-LF41 for 21 days showed no suppressive effect on LPS-induced hepatic *Tnf* mRNA and serum ALT levels. Given the important role of COX-2 in controlling upregulation of hepatic PGE_2_ levels, we hypothesized that the failure of induction of ileal COX-2 expression after 21 days treatment of H-LF41 might be accountable for the lack of preventive effect. Nevertheless, it is unclear whether components from LF41 could directly induce COX-2 expression in the ileum. On the other hand, mice treated with H-LF41 for 3 weeks had higher mRNA levels of two antimicrobial peptides (REG3γ and REG3β), TGF-β, and IL-6. Because the latter three were not affected by 10 days of H-LF41 treatment, it seems that they might have potential association with the decreased amounts of ileal DNA and COX-2 and even the lack of preventive action against LPS.

It has been well demonstrated that PGE_2_ inhibits LPS-induced expression of pro-inflammatory cytokines, such as TNF-α [29, 32]. In addition, PGE_2_ promotes LPS-activated IL-10 expression in macrophages [29, 32]. The PGE_2_-mediated regulatory effect on expression of TNF-α and IL-10 has been shown to be mediated via the EP4- and/or EP2-dependent pathways in diverse innate immune cells [32, 33], such as in hepatic Kupffer cells [30]. Consistently, we observed in the present study that oral pretreatment of H-LF41 for 10 days led to increase in hepatic PGE_2_ and aggrandizement of the hepatic IL-10 protein levels activated by LPS. In our study, EP4-specific blockade abolished LF41-mediated attenuation of the hepatic TNF-α expression, implying that contribution by the PGE_2_-EP2 pathway may be limited. Administration of recombinant IL-10 protein has been shown to improve liver function in a LPS-induced murine septic model [34]. Consistent with our study, the increased hepatic IL-10 by LF41 was also responsible for the attenuation of LPS-induced serum ALT levels. However, whether the increased hepatic PGE_2_ in LF41-administered mice could cause the facilitation of hepatic IL-10 levels in the presence of LPS remains unclear, especially in view of our finding that specific blockade of PGE_2_-EP4 pathway had marginal influence on LF41-mediated increase in hepatic IL-10 (data not shown). This might be due to the involvement of the PGE_2_-EP2 pathway in PGE_2_-mediated facilitation of IL-10 expression in the presence of LPS [32]. On the other hand, we also showed that LF41-involved augmentation in hepatic IL-10 levels was significantly diminished after the COX-2 blockade, which also abrogated LF41-mediated up-regulation of hepatic PGE_2_. Moreover, pretreatment with PGE_2_ significantly attenuated serum ALT levels and promoted hepatic Il10 mRNA levels in the presence of LPS. These largely argue against the possibility that the enhancement of IL-10 levels is regulated by other unidentified factor(s). Since in LF41-administered mice, the upregulation of hepatic PGE_2_ or IL-10 levels was controlled by COX-2, the increased COX-2 may be indirectly responsible for LF41-mediated preventive action against LPS.

The precise mechanism by which the upregulation of hepatic PGE_2_ is regulated remains elusive. We only propose here that the induced hepatic PGE_2_ may be not derived from the liver but from other organs, especially the intestine. Firstly, mice given 10 days supplement of H-LF41displayed no alteration in either COX-1 or COX-2 expression in the liver in comparison with the control mice. Secondly, 10 days of H-LF41 challenge was associated with increase in COX-2 expression and PGE_2_ secretion in the terminal ileum, and COX-2 blockade eliminated the increase not only in the terminal ileal PGE_2_ secretion but also hepatic PGE_2_ amount. Moreover, after H-LF41 challenge, IL-10 expression was observed to be upregulated in the terminal ileum but not the liver. However, the blockade of IL-10 facilitated LF41-involved upregulation of hepatic PGE_2_ and ileal COX-2 levels via a COX-2-dependent mechanism, without inducing hepatic COX-2 protein levels. However, the involvement of other mechanism, such as the reduced degradation of PGE_2_ mediated by 15-hydroxyprostaglandin dehydrogenase [33], cannot be completely excluded.

COX-2 and IL-10 in LF41-pretreated mice were required for preventing an increase in ileal TNF-α secretion and TNF-α-mediated enhancement of intestinal epithelial permeability. *Tnf* mRNA levels in LF41-fed mice was significantly up-regulated in both the lamina propria cells and epithelial cells of the terminal ileum after COX-2 blockade. However, the mechanism of increased ileal TNF-α expression after COX-2 or IL-10 blockade in mice treated with H-LF41 for 10 days remain unclear. Exposure of isolated terminal ileal tissue to LF41-generated conditioned medium led to marked enhancement of TNF-α secretion. Consistently, LGG-produced conditioned medium also showed a stimulative effect on ileal TNF-α secretion, whereas the conditioned medium from BC41 did not (**S4 Fig**). These imply that strains of *Lactobacillus* such as LF41 and LGG have an ability to induce pro-inflammatory cytokine expression in intestinal tissues if in physical contact with them. Indeed, the host’s innate cells sense microbial products, derived from either pathogenic or commensal bacteria, via evolutionarily conservative pattern recognition receptors, and trigger cellular downstream signaling cascades, which can induce production of pro-inflammatory cytokines such as TNF-α [35]. Consistent with our results, strains of lactic acid bacteria have been shown to induce high TNF-α expression in dendritic cells or blood mononuclear cells [36–37], and LGG and *Lactobacillus planetarium* NCIMB8826 do the same in human intestinal tissues in a cell culture system, if the bacterial cells or products gain access to the tissues [38]. In addition, VSL#3 in the ileal epithelial cells of a type of mouse prone to develop spontaneous ileitis is associated with increased production of TNF-α and restoration of epithelial barrier function *in vivo* [39].

Similar to its effect on the ileum, COX-2 in LF41-challenged mice also acted as a preventive role in averting enhancement of TNF-α expression in hepatic mononuclear cells (HMNCs). The enhanced TNF-α expression in HMNCs may be as a result of the increased intestinal permeability after blockade of either COX-2 or IL-10, and the lack of upregulation of hepatic PGE_2_ after either the COX-2 blockade or the co-blockade of COX-2 and IL-10. In support of this, the IL-10 blockade caused increase in both intestinal permeability and the already upregulated hepatic PGE_2_ levels associated with LF41, but did not enhanced TNF-α expression in HMNCs. Besides its pro-inflammatory features, TNF-α is able to induce apoptosis directly in diverse cells, such as hepatocytes [40]. For that reason, it seems essential for the murine host to have a precautionary mechanism against potential unwanted pressure from H-LF41 administration. This could include enhancement of hepatic and ileal TNF-α expression, which could pose a potential risk of cellular damage via induction of apoptosis. Given that PGE_2_ has the ability to resist apoptosis of intestinal epithelial cells induced by diverse stimuli [41–42], the upregulation of COX2 in response to H-LF41 might be just such a preventive reaction in the murine host.

Unlike the effect observed with COX-2 blockade, the EP4 blockade did not enhance TNF-α expression of the HMNCs and terminal ileum, which is well associated with the failure to enhance intestinal permeability. This implies that COX-2-controlled prevention against increase in the TNF-α expression is not mediated via a PGE_2_-EP4-dependent pathway, which seems contradictory to the involvement of this pathway in inhibiting hepatic TNF-α expression. In the present study, IL-10 blockade reinforced TNF-α expression in the terminal ileum but not in the HMNCs and 10 days of H-LF41 gavage had no regulatory influence on hepatic IL-10 expression. We thus hypothesize that in mice challenged with H-LF41 for 10 days, the enhanced IL-10, but not activation of a PGE_2_-EP4-dependent pathway may be required for preventing an increase in ileal TNF-α expression. This may also account for the discrepancy between involvement of EP4 pathway in the regulation of TNF-α expression in mice fed LF41 alone, and that in mice pre-fed with LF41 and administered LPS.

In the present study, the interaction of COX-2 and IL-10 in mice fed H-LF41 for 10 days and the underlying regulatory mechanism of the expression of either were not fully delineated. In LF41-administered mice, enhanced expression of COX-2 and IL-10 were found to be restricted in the epithelial cells and underlying lamina propria cells of the terminal ileum, respectively. LF41-mediated increase in COX-2 protein in the terminal ileum was further facilitated by IL-10 blockade, suggesting that excessive induction of ileal COX-2 is also prevented and controlled. As TNF-α has been shown to enhance intestinal epithelial expression of COX-2 *in vitro* [43–44], the enhanced ileal TNF-α secretion after the IL-10 blockade might have contributed to the increased ileal COX-2 protein levels. Contrast with its amplification of LPS-activated hepatic IL-10 levels, the COX-2 blockade had no regulatory influence on LF41-involved upregulation of ileal IL-10 gene expression (data not shown). This may be due to the enhanced intestinal permeability after the IL-10 blockade. Nevertheless, the ileal expression of COX-2 and IL-10 correlated well, both of which were higher in mice fed H-LF41 for 10 days, and returned to the baseline levels after 3 weeks of H-LF41 administration.

On the other hand, the mechanism of LF41-mediated up-regulation of COX-2 in epithelial cells of the terminal ileum is still unclear. Although oral challenge with UV-killed LF41 alone did not stimulate ileal COX-2 expression, co-administration of UV-killed LF41 and H-LF41 facilitated the expression associated with H-LF41 treatment alone, implicating the components derived from dead bacterial cells in LF41-mediated *in vivo* upregulation of COX-2 expression. Because there was no change in intestinal permeability after H-LF41 challenge for either 5 (data not shown) or 10 days, it seems that LF41-derived active metabolites might also contribute to the induction of COX-2 expression *in vivo*. However, LF41-derived conditioned medium alone was not found to induce COX-2 expression in intestinal epithelial cell lines such as IEC-6 and Caco-2 (data not shown). In contrast, *Lactobacillus acidophilus*-derived condition medium has been shown to induce COX-2 expression in Caco-2 cells [45]. Besides our study, there have reports about the effect of orally-challenged strains of lactic acid bacteria on the induction of intestinal PGE_2_ or COX-2 expression, or both. Healthy elderly people have shown increased amounts of fecal PGE_2_ after oral treatment with *Lactobacillus acidophilus* NCFM [46]. In a rat model of necrotizing enterocolitis, orally-administered *Bifidobacterium bifidum* OLB6378 have a stimulatory effect on ileal COX-2 and PGE_2_ expression [47]. However, unlike our results, it seems that oral administration of this strain alone does not induce COX-2 expression [47]. In spite of the preventive role of LF41-induced COX-2 in our model, COX-2 is usually regarded as a pro-inflammatory mediator, because its product PGE_2_ can facilitate influx of neutrophils from the peripheral blood into the site of infection, causing tissue swelling [28]. However, there was no typical inflammation occurred in the terminal ileum of mice fed with H-LF41 for 10 days, as evidenced by unchanged MPO activity and IL-8 levels as well as no macroscopic abnormalities in the terminal ileum. This implies that in mice treated with H-LF41 for 10 days, there might be factors that could have prevented the potential detrimental profile of the upregulated COX-2 and PGE_2_ in the intestine. Alternatively, the upregulated COX-2 and PGE_2_ in intestinal epithelial cells of LF41-treated mice might not be pro-inflammatory. Moreover, in view of the wide anti-inflammatory characteristics of IL-10, the enhanced IL-10 in LF41-administered mice might be a factor for containing the potential pro-inflammatory responses that could have been elicited by the up-regulated COX-2 and PGE_2_.

In summary, we demonstrate here that oral pretreatment of LF41 at the appropriate dose and duration significantly attenuates LPS-induced hepatic TNF-α expression and liver injury. The inhibition is essentially associated with LF41-mediated increase in hepatic PGE_2_ and LPS-activated hepatic IL-10 amounts, both under the control of COX-2 in LF41-fed mice, the expression of which is upregulated in the ileum but not the liver. Besides having repressive effect on LPS-induced inflammation, PGE_2_ inhibits the pro-inflammatory responses activated by other microbial cell wall components [32]. Thus, oral administration of LF41 at an appropriate dosage may have potential preventive application in hepatic inflammatory diseases in which LPS and other bacterial cell wall products play important pathogenic role, such as alcoholic liver disease [1, 27]. However, PGE_2_ is also implicated in the pathogenesis of diverse diseases [28], and this strain should be applied cautiously under such circumstances.

## Supporting Information

S1 Fig Influence of antibiotic treatment on fecal total bacterial 16S rRNA gene levels. q-PCR for total bacterial 16S rRNA gene amount in the feces of mice (n = 5) unmanipulated or given antibiotic formula (Ab: ampicillin (1 g/L), vancomycin (500 mg/L), neomycin sulphate (1 g/L), and metronidazole (1 g/L)) in drinking water for 4 weeks. * P < 0.05. Values are shown as mean ± SEM. Results are representative of 2 experiments with similar results.
(TIF)

S2 Fig Effect of H-LF41 administration on *Cox2*, *Il10* and *Reg3g* mRNA levels in the terminal jejuna and proximal colon. q-PCR for mRNA levels of *Cox2*, *Il10*, and *Reg3g* in the terminal jejuna and proximal colon isolated from mice (n = 8) orally treated with PBS or H-LF41 for 10 days. Results are expressed as fold change relative to PBS. **n.s.**, non-statistical difference; TJ, the terminal jejuna; PC, the proximal colon. Values are shown as mean ± SEM. Results are representative of 2 experiments with similar results.
(TIF)

S3 Fig Effect of PGE_2_ administration on mRNA levels of hepatic *Tnf* and *Il10* and serum ALT activity in response to LPS. Mice (n = 6–8) were IP challenged with PGE_2_ (200 μg/mouse in 100 μL vehicle) or its vehicle (9% ethanol) 3 h prior to LPS or PBS treatment (0.5 mg/kg body weight in 100 μL PBS; single IP injection). Hepatic *Tnf* and *Il10* mRNA levels were determined 2 h after LPS treatment **(A)**, and serum ALT levels 16 h after the treatment **(B)**. * P < 0.05.Values are shown as mean ± SEM. Results are representative of 2 experiments with similar results.
(TIF)

S4 Fig *In vitro* exposure of ileal tissue to LF41-derived conditioned medium increases TNF-α secretion. Ileal tissues were collected from mice (n = 5) and then incubated in the presence of 10% (v/v) MRS (control), LF41-derived conditioned medium (LF41-CM), BC41-CM, or LGG-CM. After 24 h incubation, TNF-α protein levels in the culture supernatant were determined by ELISA. * P < 0.05. Values are shown as mean ± SEM. Results are representative of 2 experiments with similar results.
(TIF)

S1 Table Primer sequences for real time PCR. (DOCX)
