# Supplementary figures and images for: Lactobacillus fermentum ZYL0401 Attenuates Lipopolysaccharide-Induced Hepatic TNF-α Expression and Liver Injury via an IL-10- and PGE2-EP4-Dependent Mechanism
Source: PLoS One. 2015 May 15;10(5):e0126520. doi: 10.1371/journal.pone.0126520 (PMC4433256; doi:10.1371/journal.pone.0126520)

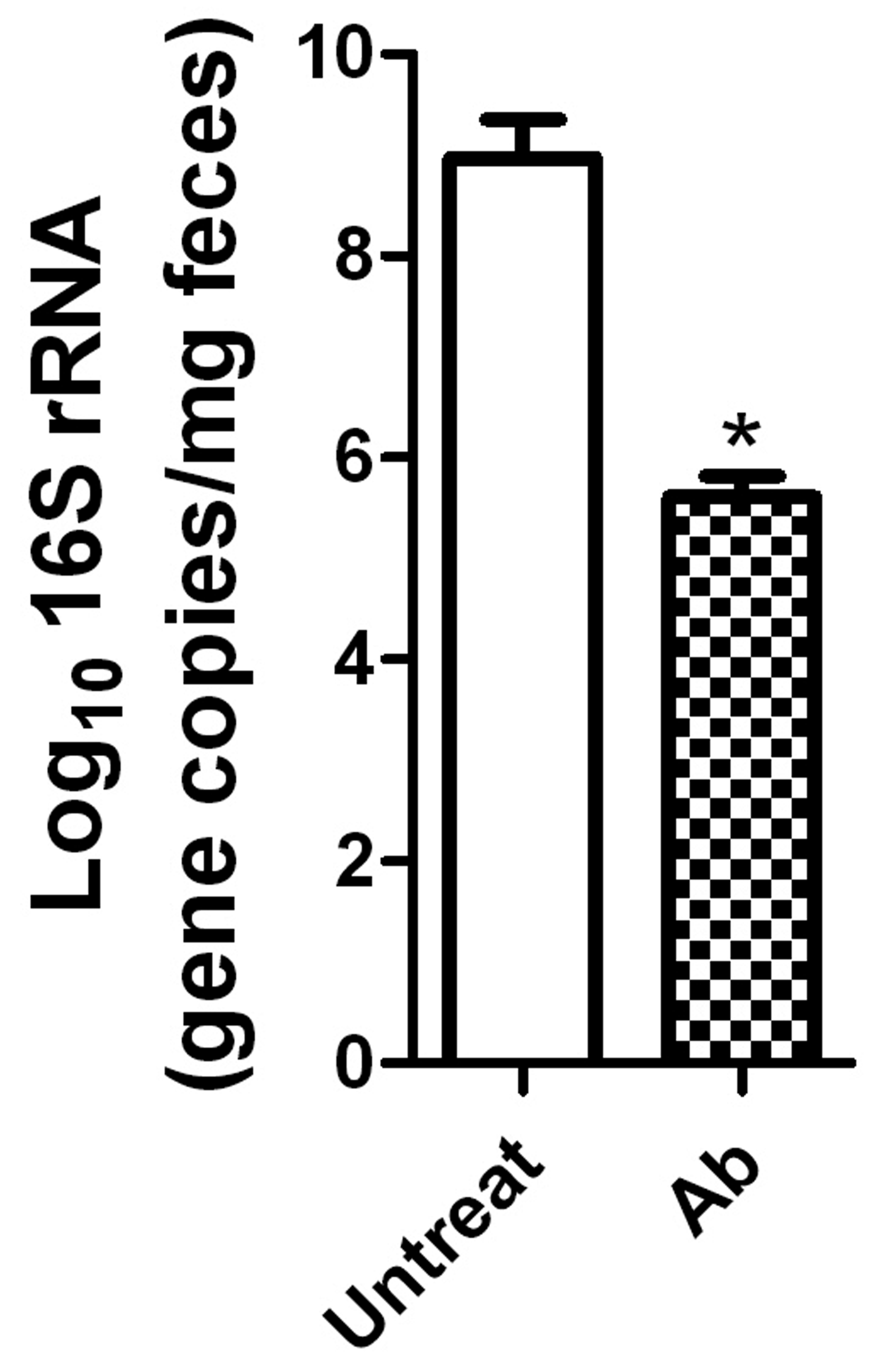

Supplement: S1 Fig — q-PCR for total bacterial 16S rRNA gene amount in the feces of mice (n = 5) unmanipulated or given antibiotic formula (Ab: ampicillin (1 g/L), vancomycin (500 mg/L), neomycin sulphate (1 g/L), and metronidazole (1 g/L)) in drinking water for 4 weeks. * P < 0.05. Values are shown as mean ± SEM. Results are representative of 2 experiments with similar results. (TIF) [file pone.0126520.s001.tif]

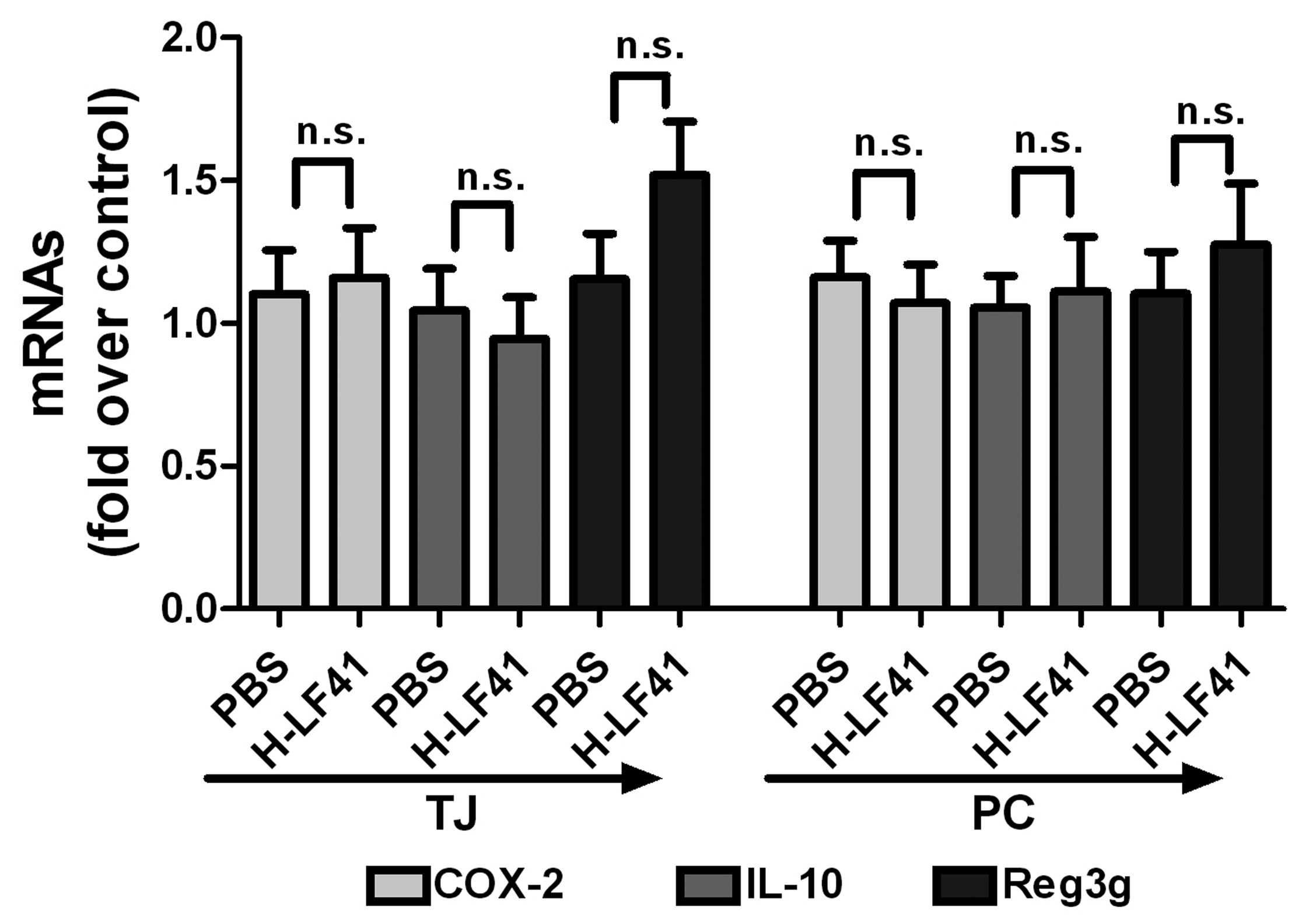

Supplement: S2 Fig — q-PCR for mRNA levels of Cox2, Il10, and Reg3g in the terminal jejuna and proximal colon isolated from mice (n = 8) orally treated with PBS or H-LF41 for 10 days. Results are expressed as fold change relative to PBS. n.s., non-statistical difference; TJ, the terminal jejuna; PC, the proximal colon. Values are shown as mean ± SEM. Results are representative of 2 experiments with similar results. (TIF) [file pone.0126520.s002.tif]

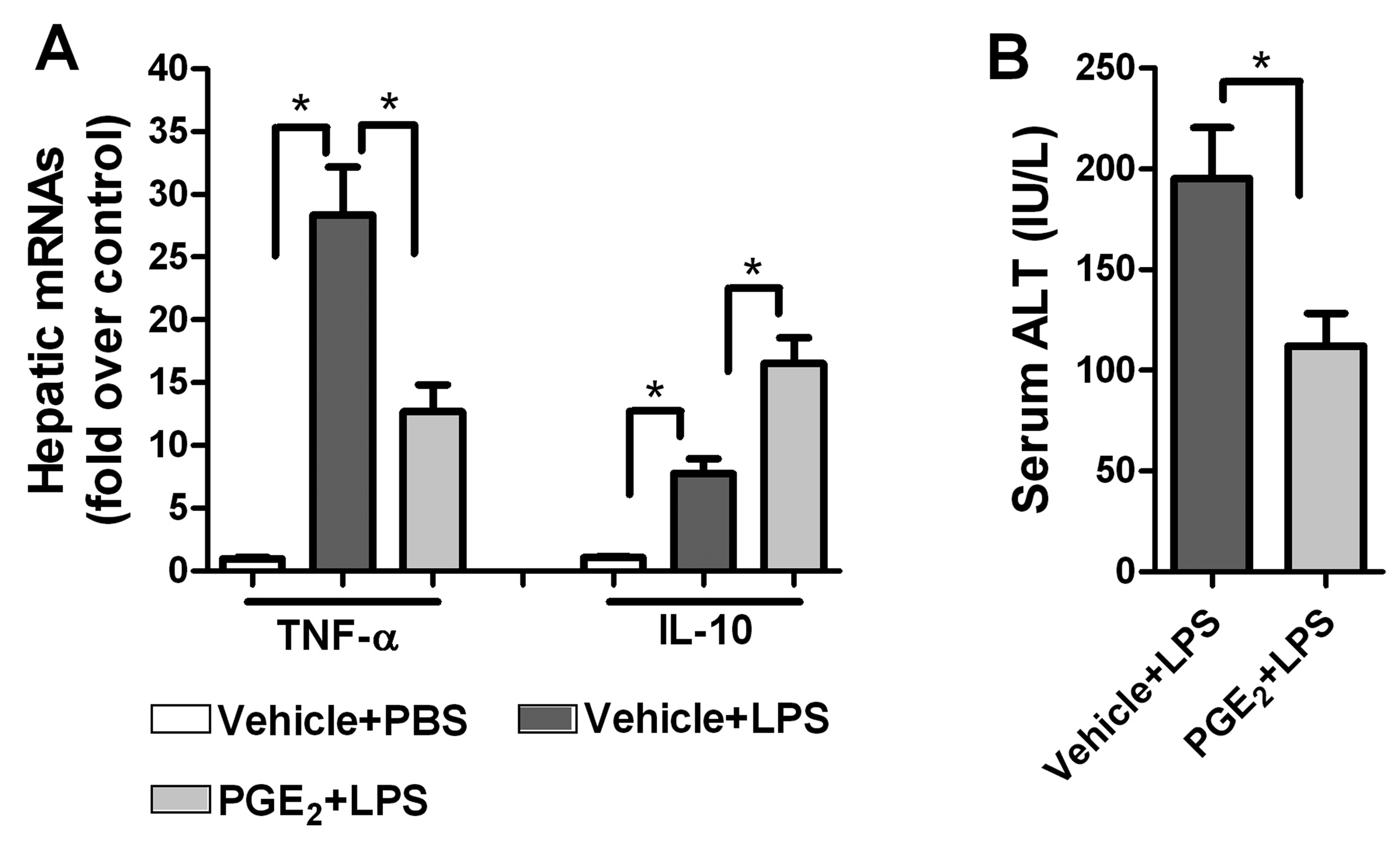

Supplement: S3 Fig — Mice (n = 6–8) were IP challenged with PGE2 (200 μg/mouse in 100 μL vehicle) or its vehicle (9% ethanol) 3 h prior to LPS or PBS treatment (0.5 mg/kg body weight in 100 μL PBS; single IP injection). Hepatic Tnf and Il10 mRNA levels were determined 2 h after LPS treatment (A), and serum ALT levels 16 h after the treatment (B). * P < 0.05.Values are shown as mean ± SEM. Results are representative of 2 experiments with similar results. (TIF) [file pone.0126520.s003.tif]

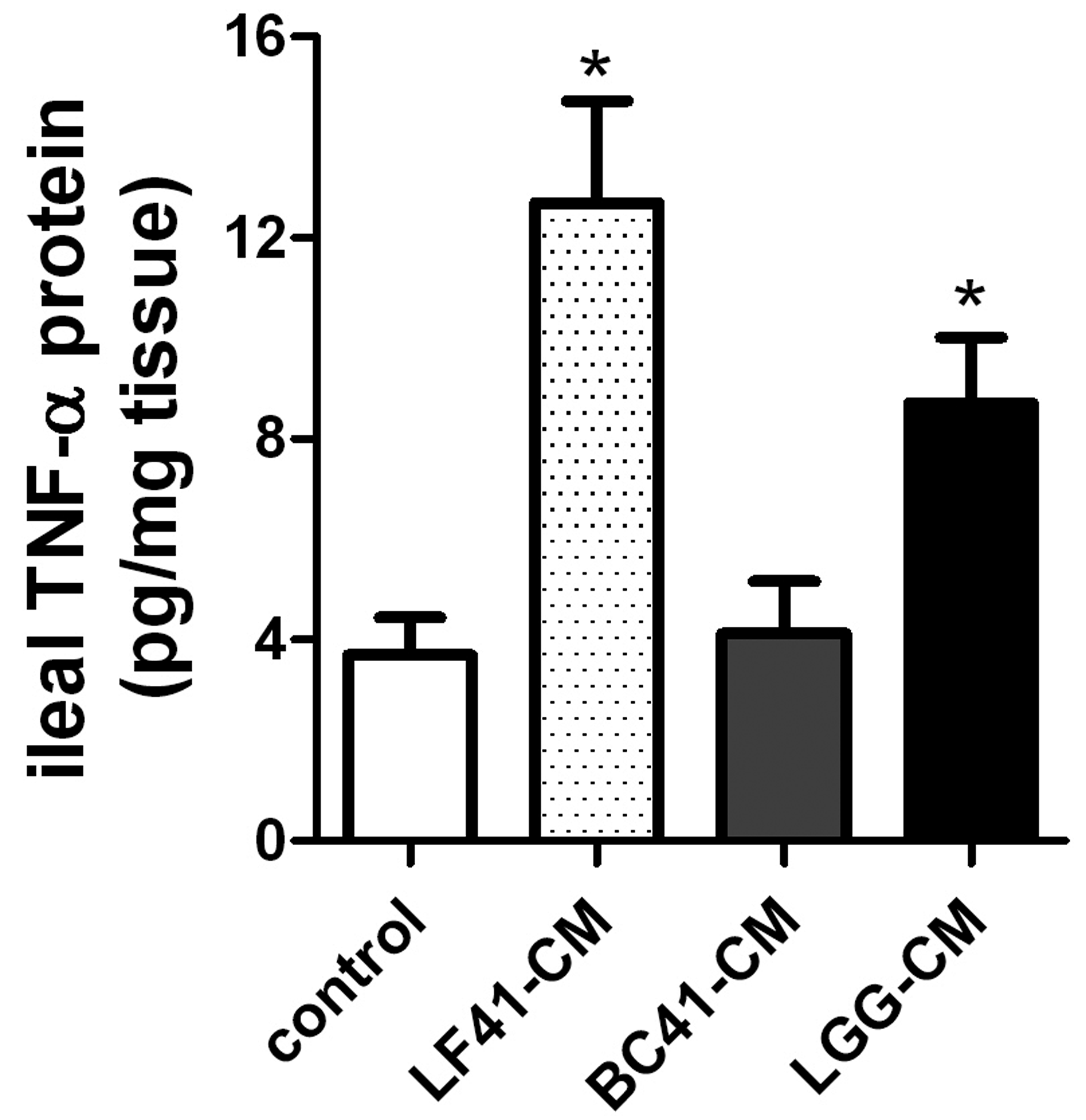

Supplement: S4 Fig — Ileal tissues were collected from mice (n = 5) and then incubated in the presence of 10% (v/v) MRS (control), LF41-derived conditioned medium (LF41-CM), BC41-CM, or LGG-CM. After 24 h incubation, TNF-α protein levels in the culture supernatant were determined by ELISA. * P < 0.05. Values are shown as mean ± SEM. Results are representative of 2 experiments with similar results. (TIF) [file pone.0126520.s004.tif]
