# Supplementary material for: Lactobacillus fermentum ZYL0401 Attenuates Lipopolysaccharide-Induced Hepatic TNF-α Expression and Liver Injury via an IL-10- and PGE2-EP4-Dependent Mechanism
Source: PLoS One. 2015 May 15;10(5):e0126520. doi: 10.1371/journal.pone.0126520 (PMC4433256; doi:10.1371/journal.pone.0126520)
Supplement: S1 Table — (DOCX) [file pone.0126520.s005.docx]

**S1** **Table.** Primer sequences for real time PCR

| **Name** | **Forward primer** | **Reverse primer** |
| --- | --- | --- |
| Mouse IL-8  Mouse TNF-α | 5’-ATGACTTCCAAGCTGGCCGT-3’  5’-ACGTGGAACTGGCAGAAGAG-3’ | 5’-TTACATAATTTCTGTGTTGGC-3’  5’-CTCCTCCACTTGGTGGTTTG-3’ |
| Mouse CCL4  Mouse IL-5  Mouse IL-4 | 5’-TTCTGTGCTCCAGGGTTCTC-3’  5’-AGCACAGTGGTGAAAGAGACCTT-3’  5’-ACAGGAGAAGGGACGCCA T-3’ | 5’-CGGGAGGTGTAAGAGAAACAG-3’  5’-TCCAATGCATAGCTGGTGATT T-3’  5’-GAAGCCCTA CAGACGAGCTCA-3’ |
| Mouse INF-γ  Mouse IL-12 | 5’-TCAAGTGGCATAGATGTGGAAGAA-3’  5’-CTTTGATGATGACCCTGTGC-3’ | 5’-TGGCTCTGCAGGATTTTCATG-3’  5’-TTTGGGGAGATGAGATGTGA-3’ |
| Mouse IL-1β | 5’-CAGGATGAGGACATGAGCAC-3’ | 5’-CAGTTGTCTAATGGGAACGTCA-3’ |
| Mouse IL-6  Mouse iNOS | 5’-TGGAGTCACAGAAGGAGTGGCTAAG-3’  5’-CAGCTGGGCTGTACAAACCTT-3’ | 5’-TCTGACCACAGTGAGGAATGTCAA-3’  5’-CATTGGAAGTGAAGCGTTTCG-3’ |
| Mouse β-actin | 5’-AGAGGGAAATCGTGCGTGAC-3’ | 5’-CAATAGTGATGACCTGGCCGT-3’ |
| Mouse COX-2 | 5’- CCAGCACTTCACCCATCAGTT -3’ | 5’- ACCCAGGTCCTCGCTTATGA-3’ |
| Mouse Reg3g | 5’-CCTTCCTCTTCCTCAGGCAAT-3’ | 5’-TAATTCTCTCTCCACTTCAGAAATCCT-3’ |
| Mouse Reg3b  Mouse TGF-β | 5’-CTGCCTTAGACCGTGCTT TC-3’  5’-GGTTCATGTCATGGATGGTGC-3’ | 5’-ATAGGGCAACTTCACCTCAC-3’  5’-TGACGTCACTGGAGTTGTACGG-3’ |
| Mouse IL-10  Mouse IL-22 | 5’-GCTCTTACTGACTGGCATGA-3’  5’-GTGGGATCCCTGATGGCTGTCCTGCAG-3’ | 5’-CGCAGCTCTAGGAGCATGTG-3’  5’-AGCGAATTCTCGCTCAGACTGCAAGCA-3’ |
